# Beyond uniformity: individual sensitivities to reward and punishment shape midfrontal-theta responses to approach avoidance conflict

**DOI:** 10.1093/scan/nsaf114

**Published:** 2025-10-31

**Authors:** Shubham Pandey, Roman Osinsky

**Affiliations:** Differential Psychology and Personality Research Unit, Institute of Psychology, Osnabrück University, Osnabrück 49076, Germany; Differential Psychology and Personality Research Unit, Institute of Psychology, Osnabrück University, Osnabrück 49076, Germany

**Keywords:** EEG, mid-frontal theta, approach, avoidance, cognitive control

## Abstract

Approach-avoidance conflict (AAC) is a core aspect of decision-making, involving competing appetitive and aversive outcomes. Given substantial individual differences in sensitivity to reward and punishment, AAC experiences likely vary across individuals. This study examined the neurocognitive mechanisms underlying AAC, focusing on these individual differences. Participants completed a task with four levels of reward and punishment probabilities (.25, .50, .75, 1), choosing to accept or reject rewards paired with potential punishments. Importantly, we did not find a uniform condition that consistently elicited maximum or minimum AAC across participants. We identified individualized high- and low-conflict conditions using three metrics: reaction time, reward rejection rate, and a composite Behavioural Conflict Index. Mid-frontal theta (MFT) power, an established marker of conflict monitoring, was significantly higher in high-conflict compared to low-conflict trials. Computational modelling revealed variability in participants’ weighting of reward and punishment probabilities, reflecting differences in participants’ sensitivity to reward and punishment outcomes. Furthermore, self-reported Behavioural Inhibition System scores predicted MFT responses, linking personality traits to AAC processing. These findings demonstrate that MFT power reflects AAC-driven cognitive control, shaped by individual sensitivities and traits. Our results emphasize the importance of personalized conflict definitions for understanding adaptive control in motivationally ambiguous contexts.

## Introduction

Cognitive control and conflict monitoring are fundamental processes that enable individuals to navigate competing motivational demands in everyday decision-making. One crucial form of conflict arises when individuals must resolve the tension between concurrently activated approach and avoidance tendencies. That is, the same behaviour involved in obtaining potential reward also potentially leads to an aversive outcome. Understanding how the brain manages approach-avoidance conflicts (AAC) has broad implications, ranging from theories of decision-making to applications in clinical settings for disorders marked by impaired cognitive control, such as anxiety and addiction (Letkiewicz et al. 2023).

Mid-frontal theta (MFT) activity, originating from the posterior midfrontal cortex (pMFC), has been consistently implicated in ­cognitive control and conflict monitoring (Cavanagh et al. 2009, Cohen and Donner 2013, Cavanagh and Frank 2014, Duprez et al. 2020, Muralidharan et al. 2023). In particular, transient increases in MFT power are thought to reflect neural mechanisms responsible for detecting conflict, signalling the need for control, and facilitating adaptive behavioural responses. Despite extensive research on MFT in simpler forms of stimulus-response conflict tasks, researches have just begun to investigate the role of MFT in the context of AAC, where motivational and affective processes strongly influence behaviour (Ullsperger et al. 2014, Lange et al. 2022, Ziebell et al. 2023).

Moreover, previous studies on conflict processing have mainly relied on pre-assumed uniform “high conflict” and “low conflict” classification (e.g. incongruent and congruent trials in stroop/flanker tasks). However, recent findings suggest that an individualized approach can better inform us about neurocognitive mechanisms of conflict processing. For instance, Pinner and Cavanagh (2017) showed that MFT power can account for varying behavioural tendencies across individuals. In another study, Lin et al. (2018) showed that individualized conflict parametrically modulated MFT power. Their study tailored conflict levels for each participant using parameters extracted from an intertemporal choice task that participants performed before the Electroencephalogram (EEG) task. With regard to AAC, Lange et al. (2022) found systematic individual differences in reward-punishment configurations that led to highest level of AAC and corresponding MFT responses. It is very likely that such differences arise from variability in stable sensitivities to reward and punishment cues and, consequently, approach-avoidance tendencies (Gray and McNaughton 2000, Corr 2004). Consequently, the point of maximal AAC is probably not uniform across individuals. Neglecting such individual differences in AAC processing may significantly diminish contrast statistics for measures of neural activity (compare, Ahn et al. 2011). In the present study, we therefore investigated the link between MFT and AAC-processing by incorporating individualized definitions of conflict conditions, thereby accounting for participants’ unique sensitivities to rewards and punishment. We use a task with multiple combinations of reward and punishment probabilities to better account for inter-individual variability in sensitivity to reinforcers. Rather than relying on pre-assumed “high conflict” and “low conflict” conditions (e.g. incongurent and congruent trials in Stroop tasks or similar tasks, fixed combination of reward and punishment here), we operationalized conflict on a participant-by-participant basis using behavioural conflict-indices such as reaction time and reward rejection rate.

## Method

### Participants, apparatus and stimuli

Forty volunteers (29 females) aged 18 to 32 years (M = 22.05 years, SD = 3.07 years) with normal or corrected normal vision took part in this study. All participants were recruited through flyer advertisements, provided their informed written consent, and reported being in good health, free of medications, and without any history of psychiatric or neurological disease.

The study was implemented using the PsyToolbox in MATLAB (MathWorks Inc.). The experimental session was conducted in a dimly lit room, with participants seated at a distance of ∼90 cm in front of a 24-inch LCD flat-screen monitor (144 Hz refresh rate and 1920 × 1080 resolution). The experimental protocol began with a brief survey collecting participants’ biographic details followed by the main behavioural task.

**Figure 1.**
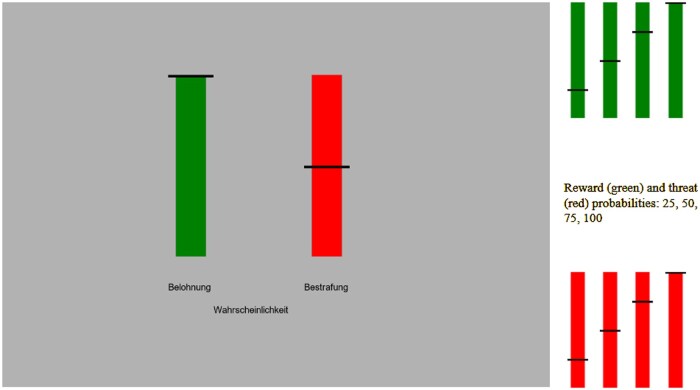
**Approach avoidance task.** The horizontal line on the bars represents the probability (.25, .50, .75, 1) of reward (green bar) and punishment (red bar) (German: Belohnung: reward; Bestrafung: punishment). In each trial, participant needed to decide whether to go for the reward under the given constellation of reward-punishment probabilities.

In each trial, two vertical bars of same size were presented on screen (see Fig. 1): a red for reward, a green for punishment. A black dash marker positioned on each bar indicated the probability of reward and punishment. The black dash could appear at one of the four equidistant positions, corresponding to probabilities .25, .50, .75, or 1, resulting in 4 × 4 = 16 reward-punishment probability combinations. Participants were informed that they could win points, which they can later convert into monetary reward of around ten Euro. As a punishment, participants heard one of three aversive sounds (90 dB) delivered through an over-the-ear headphone, randomly selected.

#### Experiment procedure

The screen background colour was set to grey [0.7 0.7 0.7]. Each trial began with a centrally presented fixation lasting 500 ms. This was followed by presentation of two vertical bars: a red one and a green one, appearing on left and right side of screen, counterbalanced across participants, for a duration of 1500 ms and equidistant from centre of screen (2.7°). We refer the green bar as *reward meter* and the red bar as *punishment* (*threat) meter*. Participants were instructed to quickly press *left* or *right* arrow key button to accept or decline the reward. The side of the reward vs. punishment meter, as well as the mapping of the accept vs. decline buttons, was always aligned and counterbalanced across participants. The maximum time to response was same as the time of stimulus display, and the stimulus stayed on screen for entire duration of 1500 ms irrespective of participant response.

Stimulus display was followed by a feedback for 700 ms where a box was shown. There were three possible participant responses: accept the reward, decline the reward, or make no response within the allotted time. If the participant accepted the reward, one of four outcomes could occur based on the predefined probabilities: (1) a sole reward, (2) a sole punishment, (3) both reward and punishment, or (4) neither reward nor punishment. In a reward only trial, they were shown a green box with “+5 points” superimposed on it. In a both rewarding and punishing trial, they saw a half red-half green box with “+5 points.” Punishment-only trials were indicated by a red box. Trials with neither reward nor punishment yielded no feedback. The potential outcome of a given trial was predefined, taking the reward and punishment probabilities into account. Winning a reward was solely determined by reward probability, e.g. a reward probability of 1 led to reward on all such trials, while a reward probability of .5 led to a reward on only 50% of such trials. The same principle applied to punishment probability and actual punishment delivery. In case participants declined the reward, they received neither reward nor punishment, as indicated by a black box. If no response was made within the response window, participants forfeited the reward but could still receive punishment (red box), depending on the predetermined trial outcome. If such a trial did not contain punishment, no feedback was shown. Following the feedback, there was an inter-trial interval of mean 1500 ms drawn from a pseudo-Gaussian distribution ranging from 1000 ms to 2000 ms.

The experiment consisted of eight blocks, each comprising of 64 trials. Each of the sixteen reward-punishment probability combinations was represented by four trials in a block, totalling 32 trials per condition across the experiment. Trials presentation was pseudo-randomized to ensure that no condition was repeated in subsequent trials. After each block, participants were shown their cumulative reward points. Each participant underwent an initial practice session of 4 trials for each of sixteen conditions to familiarize themselves with the task. The task roughly took 40 minutes to complete, with an additional 60 minutes for EEG preparation and hair wash.

#### Research design and data analysis

To address our main research question, we aimed to contrast conditions of maximum conflict with those of minimum conflict. We implemented two distinct approaches for defining these conditions, as described in detail below.

In the *uniform* approach we assumed the same task condition definitions across all participants. Specifically, we posited that the 50% reward—50% punishment condition would elicit highest whereas the 100% reward—25% punishment or 25% reward—100% punishment condition would represent lowest AAC.

In the *individualized* approach, we accounted for inter-individual variability by identifying personalized conditions of maximum and minimum conflict. These were determined based on participant’s reward rejection rates, reaction times (RTs), or a combination of both. In each method, we selected the top three and bottom three conditions to represent high and low AAC, respectively. We picked three conditions instead of one condition for two reasons: first, it increased total available trials for EEG analysis, and second, more importantly, for several participants there were more than one condition with 0% or 100% reward rejection rate. For the sole RT based approach, we assumed the three conditions with highest mean RT as maximum conflict condition, and the three condition with lowest RT as minimum conflict conditions. For the sole rejection-rate based approach, we assumed the three conditions with reward rejection-rate closest to 50% as highest conflict conditions, and the three conditions with rejection-rate closest to 0 or 100% as minimum conflict conditions. However, we found that, for some participants, there were more than three conditions with same reward rejection rate (e.g. 0% for all for conditions whenever punishment probability was 0.25). For these cases, we randomly chose three conditions out of all qualifying conditions.

In our third individualized approach, we combined RT and reward rejection rate to create a new metric reflecting the individual degree of experienced conflict. We used this metric to identify *individualized* conditions of maximum and minimum conflict. We took this approach based on previous findings, indicating the that inclusion of reaction times along with choice data (rejection rates) improve the accuracy of metric estimation (Prerau et al. 2009, Ballard and McClure 2019, Zorowitz et al. 2019). Notice that rejection rate exhibits a V-shaped distribution, with maximum conflict occurring around 50% acceptance/rejection rate. Conflict increases as the rejection rate moves from either extreme (0% or 100%) toward 50%. Therefore, we transformed rejection rate into a linear metric, called *deviation* using the formula.


$$Deviation\;=\;0.5\;-\;\left|0.5\;-\;Reward\;rejection\;rate\right|$$

The deviation score ranges from 0 (minimal conflict) to 0.5 (maximal conflict). Accordingly, both RT and *deviation scores* are expected to linearly relate to individualized conflict. In the next step, we combined these two metrics, and computed a composite score, named “Behavioural Conflict Index” as follows:


Behavioural Conflict Index (BCI) = Reaction time × (Deviation +ε)

where *ε* was set to 0.01 to prevent behavioural conflict index (BCI) becoming 0 in case deviation was 0 (i.e. when reward rejection rate was 0% or 100%). This way, we calculated BCI scores for each of the 16 AAC conditions for each participant and picked conditions with maximum and minimum scores.

All analyses were performed in Matlab (The MathWorks, USA, version 2023 b) using custom written code. Statistical tests were performed in JASP software (Love et al. 2019). We report partial eta square (*η_p_^2^*), and Cohen’s d (*d*, mean of the difference scores divided by their standard deviation) as effect size measures. For correlation, we report Pearson correlation coefficients, *r*. Mean and standard deviation are reported with usual notation as *M* and *SD* respectively.

#### Questionnaire

Before the start of experiment, participants completed the German version of Reinforcement Sensitivity Theory of Personality Questionnaire (RST-PQ; Corr and Cooper 2016, Pugnaghi et al. 2018). Reinforcement Sensitivity Theory (Gray and McNaughton 2000, Corr 2004) posits three major neurobiological systems: the Behavioural Approach System (BAS), which promotes approach behaviour toward appetitive stimuli; the Fight–Flight–Freeze System (FFFS), which facilitates active avoidance of aversive stimuli; and the Behavioural Inhibition System (BIS), which acts as a supervisory conflict-detection mechanism. The BIS is considered to be superordinate to both BAS and FFFS and is specifically activated by motivational conflicts, especially those arising from simultaneous and opposing approach and avoidance tendencies. Based on this framework, we hypothesized that BIS scores would positively correlate with mid-frontal theta (MFT) power in response to AAC.

#### EEG data recording and processing

EEG data were recorded with a 500 Hz sampling rate and a 250 Hz low-pass and 0.016 Hz high-pass filter, using the 64 channel standard actiCAP system, BrainAmp Amplifiers and BrainVision Recorder software (all Brain Products, Gilching, Germany). The reference electrode and the ground electrode were placed at positions FCz and AFz, respectively. Off-line processing was conducted with BrainVision Analyzer 2 software. An independent component analysis (ICA) was applied for detection and removal of ocular artefacts. EEG data were then re-referenced to the average of all electrodes, and the former reference at FCz was reinstated as a new data channel. The continuous EEG data were separated into 2500 ms segments around the onset of the bars-stimulus (−1000 to 1500 ms). Trials exhibiting voltage steps >50 µV/ms, differences >400 µV within 600 ms, or low activity defined by < 0.5 µV within 100 ms were rejected from further analyses. The remaining trials were used for the time-frequency analysis and for the Generalized Eigen-Decomposition (GED; Cohen 2022), conducted using MATLAB R2020a (MathWorks).

#### Time-frequency analysis

For the time-frequency analysis, a wavelet transformation was applied on single-trial data using 30 complex Morlet wavelets, e^i2πft^e^-t2/(2s2)^, where t is time, f is frequency (ranging from 1 to 30 Hz in 30 logarithmically spaced steps), and s is the width of each frequency band, which is defined by n/(2πf) (with n logarithmically increasing from 4 to 7). Frequency specific power at each time point (t) was defined as the squared magnitude of the resulting analytic signal (Z) as real [Z(t)^2^] + imaginary [Z(t)^2^]. Resulting power levels were dB-baseline corrected per frequency layer using the average power of all trials within the −800 to −300 ms baseline time window. We conducted cluster-based permutation tests (Maris and Oostenveld 2007). We used difference of maximum and minimum conflict condition data from channel FCz, cluster-forming thresholds of *P *= .001, computed 1000 iterations. This yielded a significant region on time-frequency plot with an approximate time window from 500 to 1100 ms. MFT was extracted as mean power within 4 Hz to 8 Hz at FCz.

#### Generalized Eigen-decomposition and source reconstruction

**Figure 2.**
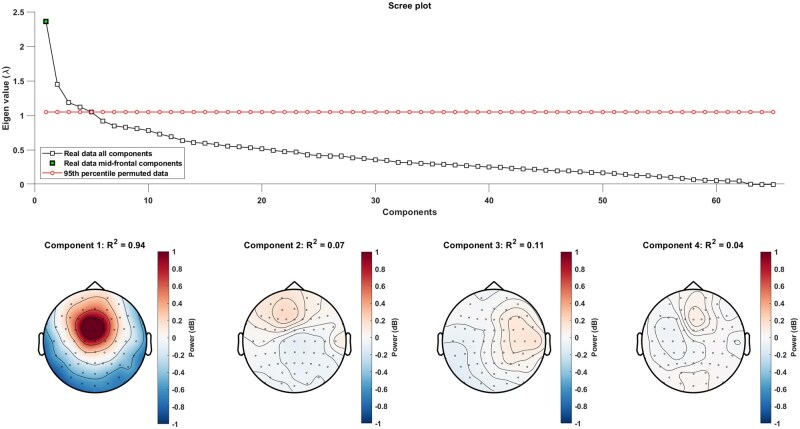
Significant components identified with GED for one model participant.

We performed GED to separate different sources for theta band activity and then singled out components showing a midfrontal topography. This procedure is based on the approach taken by (Zuure et al. 2020). The GED was designed to yield filters that maximally differentiate between broadband EEG activity and theta band activity. Therefore, unfiltered but z-scored single-trial EEG activity was used for computing a reference matrix, whereas the signal matrix was computed using band-pass-filtered (4–8 Hz) and z-scored single-trial activity. Both matrices are based on the time window of 0–1000 ms around stimulus onset. Given the 65 data channels, the GED extracted 65 components per participant. The significance of these components was assessed using permutation tests. A null distribution of eigenvalues was generated by shuffling theta band filtered and unfiltered broadband activity time series 1000 times. To correct for multiple comparisons, the 95th percentile of all generated eigenvalues was used for significance testing (red line in Fig. 2) (Hayton et al. 2004). Components whose eigenvalues were not significantly greater than the eigenvalues from the randomly shuffled data (not exceeding a threshold of *α *= 0.05) were excluded from further analyses.

Next, topographical maps were computed for each component. These topographies were compared with a prototypical template (Gaussian centred at FCz) reflecting midfrontal topographical activity (Zuure et al. 2020). Components exhibiting shared spatial variance with the midfrontal template less than *R^2^* < 0.6 were excluded from further analysis. Last, the time series activity of each component was computed by multiplying the eigenvector with the EEG time series activity and then applying a time-frequency decomposition using the same parameters for a wavelet transformation as described for the previous time-frequency analysis. This procedure extracted a final set of GED components and their time-frequency activity for each participant (Zuure et al. 2020). Statistical analyses were performed based on the resulting component time-frequency activities.

## Result

### Behavioural results

#### Reaction time and reward rejection rate

**Figure 3.**
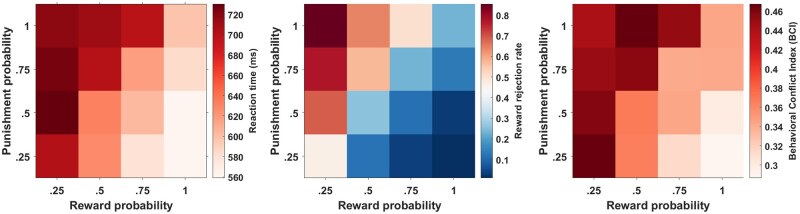
The reaction time, the reward rejection rate, and the BCI as a function of reward and punishment probability.

For RT, rejection rates and BCI scores, we performed separate two-way repeated measure ANOVAs with the two factors *reward probability* (.25, .5, .75, 1) and *punishment probability* (.25, .5, .75, 1). For RT we observed a main effect of reward probability, *F* (3, 117) = 43.22, *p* < .001, *η_p_^2^* = 0.52; a main effect of punishment probability, *F* (3, 117) = 18.59, *p* < .001, *η_p_^2^* = 0.32; and an interaction effect of both factors, *F* (9, 324) = 5.84, *p* < .001, *η_p_^2^* = 0.13 (Fig. 3). Similarly, for reward rejection rate we observed a main effect of reward probability, *F* (3, 117) = 145.62, *p* < .001, *η_p_^2^* = 0.79; a main effect of punishment probability, *F* (3, 117) = 128.95, *p* < .001, *η_p_^2^* = 0.77; and an interaction effect of both factors, *F* (9, 324) = 7.36, *p* < .001, *η_p_^2^* = 0.15. Consequently, for BCI scores we also observed a main effect of reward probability, *F* (3, 117) = 11.93, *p* < .001, *η_p_^2^* = 0.23; a main effect of punishment probability, *F* (3, 117) = 6.06, *p* < .001, *η_p_^2^* = 0.13; and an interaction effect of both factors, *F* (9, 324) = 2.87, *p* = .003, *η_p_^2^* = 0.06.

The main effects revealed that higher reward probabilities were associated with faster reaction times and lower rejection rates, whereas higher punishment probabilities were associated with slower reaction times and higher rejection rates. In other words, the likelihood of winning points facilitated faster and more accepting decisions, while the likelihood of punishment induced slower and more rejecting decisions. Given the relatively large number of conditions and factor-level combinations, interpreting the interaction terms was less straightforward. Conducting exhaustive pairwise comparisons (120 in total) would be statistically impractical. Separate ANOVAs examining one factor while holding the other constant (see Supplementary) did not reveal a clearly identifiable source of the interactions. These results suggest that our task design was effective in inducing varying level of approach-avoidance conflict, the interaction effects further suggest that participants must have considered both reward and punishment probabilities. At the inter-individual level, we observed that response trajectories became more complex and varying as approach and avoidance motivations equals, such as in conditions like .5 –.5 or .75 – .75. However, we did not observe a clear-cut pattern in mean behavioural indices that could indicate uniform conditions of maximum and minimum AAC across participants.

### EEG results

#### Time frequency analysis

**Figure 4.**
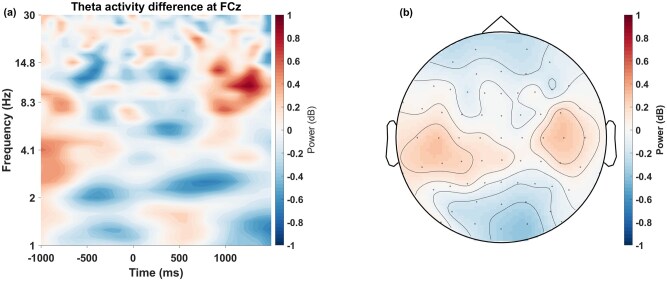
Time-frequency plot for the contrast of uniform maximum (0.5-0.5) and minimum AAC (1-0.25) condition and a topographical map of theta (4–8 Hz) power differences for this contrast in the 500–1100 ms time-window.

With our first approach of uniform conditions of maximum and minimum AAC, we did not find a time-frequency region of significant condition contrast in permutation and cluster testing (Fig. 4). Furthermore, when we compared MFT power (4–8Hz) between uniform maximum and minimum conflict conditions in a time window of 500–1100 ms, we did not find a significant difference (see Table 1 for statistics).

**Table 1.** MFT from maximum and minimum conflict conditions based on Uniform and Individualized approaches of computing AAC, and corresponding statistics.

Analysis type		MFT (dB) maximum conflict		MFT (dB) minimum conflict		Whether signifiant region after permutation-cluster correction	Comparison of maximum vs. minimum conflict			Correlation of differential power with corresponding GED components time series		Correlation of differential power with BIS score	
		M	SD	M	SD		t(39)	*p*	*d*	*r*	*p*	*r*	*p*
**Uniform approach**	**1–0.25 min**	0.32	0.92	0.32	0.92	No	0.05	0.96	0.01	0.35	0.02	0.23	0.15
	**0.25–1 min**	0.32	0.92	0.60	1.03	No	0.06	0.94	0.01	0.30	0.06	0.23	0.15
**Individualized approach**	**Rate based**	0.81	1.01	0.32	0.78	No	4.42	<.001	0.70	0.61	<.001	0.35	0.02
	**RT based**	0.92	0.97	0.27	0.82	Yes	6.43	<.001	1.01	0.62	<.001	0.20	0.20
	**BCI based**	0.84	1.00	0.28	0.81	Yes	5.48	<.001	0.86	0.67	<.001	0.35	0.02

We considered 0.5–0.5 condition as uniform condition of maximum conflict while two conditions, 1–0.25 and 0.25–1, as uniform condition of minimum conflict. Here, first value represents probability of reward while second value represents probability of punishment.

**Figure 5.**
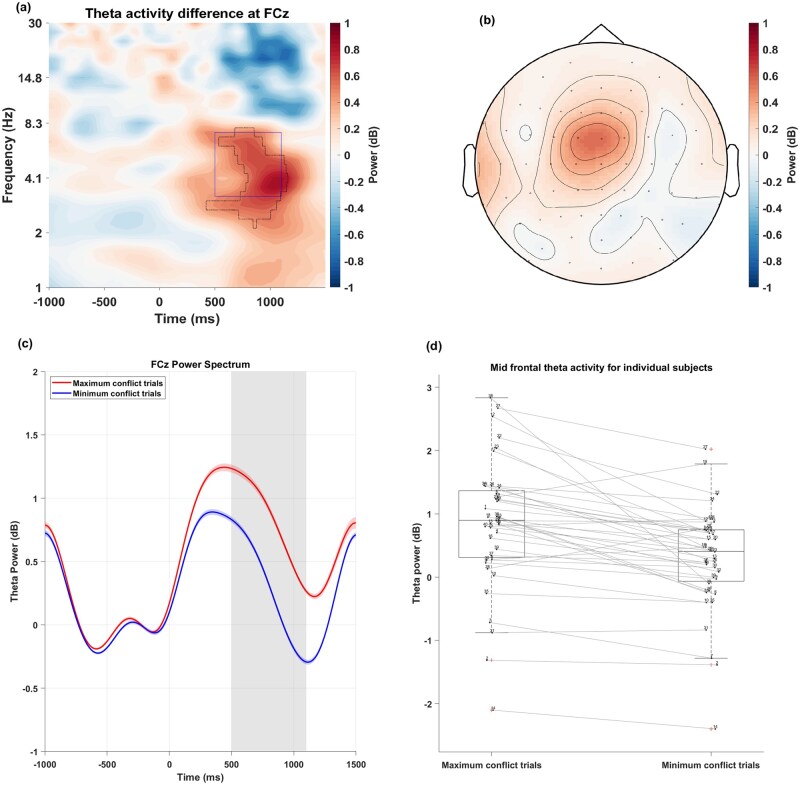
(a) Difference between theta activity of maximum and minimum conflict conditions based on individualized conflict definition according to BCI scores, averaged across participants, and corresponding topographical map at 4–8 Hz, 500–1100 ms. 0 ms refers to the onset of the bars-stimulus. The dotted line represent significant region after permutation testing and cluster correction. The rectangle represent the window used to compute topographical plot. (b) The topographical plot of corresponding time window. (c) The mid-frontal theta activity change over time. (d) The distribution of change in theta activity for individual participants.

However, the individualized approach of conflict definition based on BCI revealed significantly stronger MFT power in the maximum conflict conditions compared to the minimum conflict ­conditions, *t*(39) = 5.60, *p* < .001, *d *= 0.92 (Fig. 5, Table 1). The topo­graphy of this theta effect was centred at mid-frontal (FCz) site. Similarly, individualized approach based on RT and reward ­rejection rate also yielded significant difference between MFT of maximum and minimum conflict condition (Table 1). However, there was no significant region after permutation-cluster correction in rate based approach.

#### Source separation

**Figure 6.**
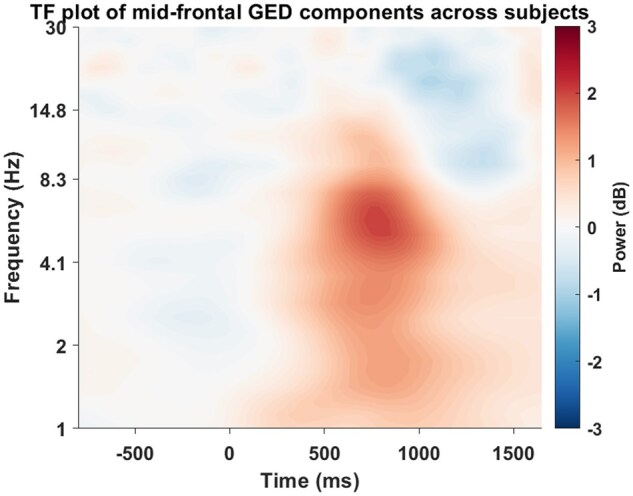
Component time-frequency activity of GED midfrontal components averaged across subjects based on BCI.

We applied a GED that extracted 65 components per participant. Based on permutation testing, only those components exceeding the significance threshold (*α*  =  0.05) were retained (M = 3.13, SD = 1.96). Next, only components showing midfrontal topographies were retained. This procedure revealed more than one GED components for 60% of the participants, and no significant component for 5% of the participants (M = 1.75, SD = 0.89). The time-frequency activity of these extracted components shows an increase in theta activity at FCz (Fig. 6). We found strong correlations between difference in MFT power of maximum and minimum conflict trials of real data based on BCI and differential MFT from extracted GED sources, *r *= 0.68, *p* < .001. We did not find a significant correlation between differential MFT from uniform approach and differential MFT from GED (Table 1). These results highlight that the increase in theta activity in individually defined maximum conflict condition compared to minimum conflict condition has mid-frontal origin.

**Figure 7.**
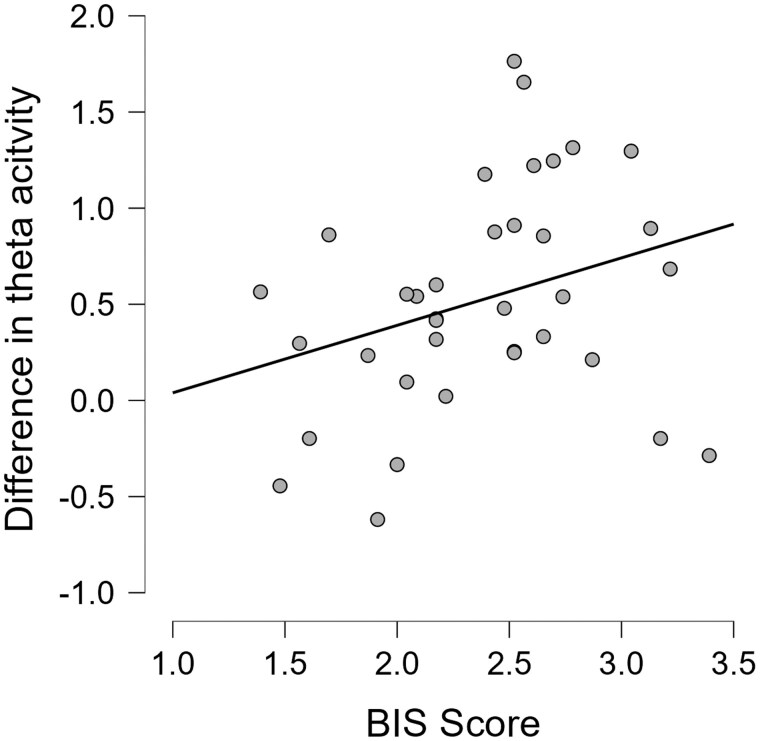
Correlation between BIS score and differential MFT based on BCI.

We found a moderate correlation between BIS score and differential MFT based on BCI (Fig. 7), *r *= 0.35, *P *= .037 suggesting that people having higher BIS score were more responsive to AAC, as reflected in their mid-frontal theta activity. However, we did not find such significant correlation with MFT when applying a uniform definition of conflict (Table 1).

#### MFT power tracks subjective conflict

**Figure 8.**
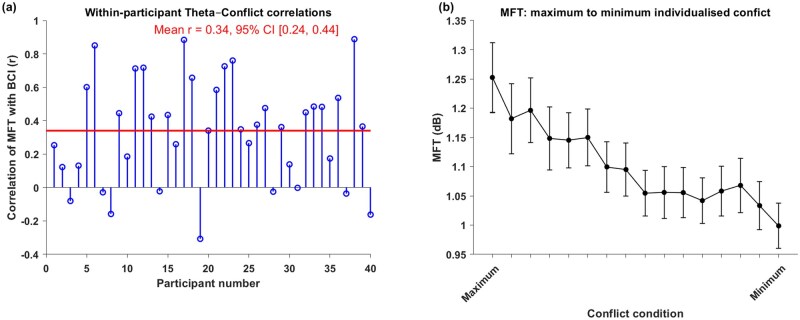
MFT power tracks subjective conflict. (a) Within-participants correlation of MFT with conflict score (BCI); (b) Gradual decrease of mid-frontal theta (MFT) power from maximum to minimum individualized conflict.

We also examined whether mid-frontal theta (MFT) power continuously tracks subjective conflict. To this end, we computed within-participant correlations between the BCI and MFT power across the 16 conditions (Fig. 8a). The correlations were significantly positive, indicating that higher subjective conflict was associated with stronger MFT power. The mean Fisher z-transformed correlation was *r *= 0.358, which was significantly greater than zero, *t*(39) = 6.63, *p* < .001 (one-tailed). To further test this relationship, we fitted a linear mixed-effects model predicting subjective conflict (BCI) from MFT power, including a random intercept for participants. The model revealed a significant positive effect of MFT power on conflict, *b *= 0.92, SE = 0.12, 95% CI [0.69-1.15], *t*(638) = 7.85, *p* < .001. The random intercept variance across participants was 0.025 (SD = 0.16), and model fit indices were AIC = –290.0 and BIC = –272.2. Together, these results demonstrate that MFT reliably tracks subjective conflict across conditions and participants.

In a next step, we leveraged participants’ subjective conflict experiences to order all 16 conditions from highest to lowest ­conflict. For each participant, conditions were sorted based on their individual BCI scores, such that the first condition represented maximum conflict and the sixteenth condition represented minimum conflict. This procedure allowed us to average MFT power across participants while preserving individual differences in conflict experience (see Supplementary for condition order for each participant). The resulting analysis revealed a gradual decrease in MFT power from maximum to minimum conflict (Fig. 8b), providing further evidence that MFT systematically tracks subjective conflict.

## Discussion

This study examined neural mechanisms of AAC processing by analysing MFT activity across varying combinations of reward and punishment probabilities. Most importantly, we observed increased MFT power in high- compared to low-AAC conditions, but only if conditions were defined individually based on behavioural indices of conflict. We also find that MFT power tracks subjective conflict. Computational modelling (see supplementary) indicated that participants integrate both reward and punishment information in decision-making, but participants weigh these factors differently.

### Mid-frontal theta and conflict monitoring

The observed increase in MFT power during the high-AAC condition aligns with existing evidence linking frontal theta activity to conflict monitoring and cognitive control processes. MFT activity of the pMFC has been identified as a neural marker of conflict detection and the recruitment of control resources (Cavanagh and Frank 2014, Lange et al. 2023). In the context of our approach-avoidance task, the individualized high-AAC conditions likely elicited heightened competition between approach and avoidance drives, necessitating increased monitoring and engagement of control mechanisms. This supports the notion that MFT activity reflects the dynamic regulation of behaviour under conditions of uncertainty or competing goals (e.g. Nigbur et al. 2011). Furthermore, in our study MFT effects persist for a rather lengthy window (up to 1200 ms) after stimulus onset, supporting the notion that these oscillations not only reflect conflict detection but also continuous deliberation and resolution between response options (Levy et al. 2023).

### Individual difference in approach avoidance sensitivities

Our results strongly indicate that individual differences in approach-avoidance sensitivities play a crucial role in conflict processing, highlighting the importance of individualized rather than predefining uniform conditions. Our findings demonstrate that when AAC was defined uniformly, MFT activity did not ­differentiate between maximum and minimum conflict trials across participants. This suggests that predefined conditions may not accurately capture participants’ actual experience of conflict, which is also supported by our behavioural data (i.e. decision time and choice rates). Instead, when conflict was defined individually based on reaction time and reward rejection rate, MFT power increased during high-conflict trials, indicating greater cognitive control demands. This shift underscores the idea that individuals weight reward and punishment information differently in decision-making, integrating both factors but assigning unique importance to each. For example, some participants may perceive a 50% - 75% reward-punishment pairing as more conflicting than a 75% to 50% pairing, depending on their individual risk tolerance, decision strategies, or more fundamental personality factors.

Our results add to literature that suggests that MFT signals of the pMFC not only reflect the need for control in “cold” cognitive processes (like the processing of simple stimulus response conflict) but may also partake in the resolution of more complex motivational inconsistencies, which are subject to strong individual differences and can be theoretically framed by the Reinforcement Sensitivity Theory of personality (RST; Gray and McNaughton 2000, Corr 2004). According to the RST, behaviour in a given situation is mainly driven by the interplay of three independent systems of a conceptual nervous systems. The BAS is sensitive to appetitive cues and activates approach behaviour. The FFFS is sensitive to aversive cues and activates avoidance behaviour. The BIS receives its input from both the FFFS and BAS is activated by motivational conflicts in general and approach-avoidance conflicts in particular. Its main function is to inhibit any ongoing behaviour and resolve conflict by increasing vigilance and information-seeking behaviour.

Our findings suggest that MFT signals of the pMFC may track subjective valence/appraisal of the reward and punishment, and thus be an important functional component of the BIS. First of all, according to the RST, the level of conflict detected by the BIS is mainly driven by an interaction of input parameters to the BAS and FFFS (e.g. the objective probabilities of potential rewards and punishments in a given situation) and the general sensitivities of these two upstream systems. When inputs from BAS and FFFS to the BIS system are identical, an individual will experience AAC only when reward and punishment are equally likely. In contrast, if input from one of the two systems to BIS is dominant, it would need a higher probability of the respective reinforcement/punishment to have an identical FFFS and BAS inputs to the BIS. The fact that we did not observe a conflict effect on MFT amplitude across participants when comparing uniformly predefined conditions but only when defining conditions on an individual basis, perfectly aligns with this assumption of BIS activation in the RST. Moreover, MFT could be a functional component of BIS as we found a significant correlation between self-reported BIS scores in the RST-PQ and MFT. Notably many of the RST-PQ BIS items refer to stable tendencies of experiencing anxious apprehension and/or arousal. Our study is therefore consistent with prior works reporting a ­relation between MFT and individual differences in trait anxiety (e.g. Cavanagh and Shackman 2015, Osinsky et al. 2017, Schmidt et al. 2018). Transient increases in MFT are also related to the inhibition of automatic approach and avoidance behaviour (Cavanagh et al. 2013, Swart et al. 2018). The BIS also modulate this automatic approach and avoidance behaviour according to RST. Finally, the BIS is thought to bias behaviour toward avoidance, in case of a non-resolvable approach-avoidance conflict, and studies have linked MFT response during AAC to this avoidance-biasing function of the BIS (Schmidt et al. 2018, Ziebell et al. 2023).

### Finding individualized conflict conditions

In our study, we employed three distinct individualized approaches to identify trials of maximum and minimum AAC: one based on reward rejection rate, one based on reaction time (RT), and third- a composite metric BCI that integrates both RT and rejection rate. Our results indicate that the RT-based and BCI-based approaches are particularly effective in capturing individualized AAC.

However, we propose that an integrated metric like BCI can provides a more robust measure of individualized AAC by accounting for both decisional hesitation and outcome variability. Importantly, our analysis showed that for some participants, the highest RTs occurred in conditions that did not align with their highest behavioural uncertainty (i.e. conditions with reward acceptance/rejection rates close to 50%). Conversely, in other cases, the lowest RTs were observed in conditions that were not associated with minimal uncertainty (i.e. reward rejection rates close to 0% or 100%). This dissociation underscores a critical limitation of relying solely on either RT or rejection rate as a proxy for conflict. While RT captures the cognitive demand associated with conflict, reward rejection rate reflects the behavioural manifestation of decisional uncertainty.

Our recommendation of BCI based approach is consistent with prior research that emphasizes the value of multidimensional behavioural indices for capturing latent cognitive processes. For example, studies have shown that combining RT and choice behaviour enhances the sensitivity of computational and behavioural models in decision-making contexts (Prerau et al. 2009, Ballard and McClure 2019, Zorowitz et al. 2019). In light of these findings, we advocate for the adoption of composite indices, such as the BCI, in future studies of approach-avoidance conflict, particularly when the goal is to capture individual variability in conflict processing with greater fidelity.

### Future directions

In our results we interpret the stimulus-locked increase in mid-frontal theta as an index of conflict monitoring, however, alternative mechanisms may also have contributed. For instance, mid-frontal theta has been linked to uncertainty and expectancy violation, raising the possibility that the observed effect may reflects participants’ ­uncertainty about the trial outcome rather than conflict. Another explanation is that individual differences in the subjective valence/salience of certain classes of rewards and punishments could drive theta variability. Since we did not directly measure subjective salience/valence (e.g. by self-reported ratings of punishment and reward stimuli) and only used one type of reward (points convertible to money) and one type of punishment (unpleasant sounds), our study is not suited to address this issue. Punishment expectancy is another process that could also lead to enhanced theta power independent of conflict. Furthermore, it is possible that prolonged response times, reflecting slower evidence accumulation, partially account for the effect. Finally, theta increases might index anticipatory control processes associated with preparing to avoid negative outcomes, rather than the simultaneous co-activation of competing responses. Although in our supplementary analysis, we show that our results are not driven by anticipation of punishment or longer reaction time, these alternative perspectives underscore the need for future work that manipulates uncertainty, outcome valence, and temporal dynamics more directly to disentangle their contributions to mid-frontal theta. A greater understanding of AAC can yield important insights of practical relevance in the field of maladaptive avoidance, thereby helping to build a bridge between experimental and applied research (Garcia-Guerrero et al. 2023). Future research may further explore how personality traits influence individual sensitivities to approach, avoidance, the conflict thereof and corresponding neural responses. Future studies should incorporate individual measures rather than relying solely on predefined conflict conditions. Moreover, further research may further explore connectivity between midfrontal and parietal regions to understand how theta and beta-related neural mechanisms interact in conflict resolution.

Conflict of interest: The authors declare that they have no competing interests.

## Supplementary Material

nsaf114_Supplementary_Data

## Data Availability

The data, data summary, and code used are available through OSF: https://osf.io/95v8j/overview?view_only=fa5062a33d4e4ab7a4488ab3df1ca050.
