# Supplementary material for: Beyond uniformity: individual sensitivities to reward and punishment shape midfrontal-theta responses to approach avoidance conflict
Source: Soc Cogn Affect Neurosci. 2025 Oct 31;20(1):nsaf114. doi: 10.1093/scan/nsaf114 (PMC12704425; doi:10.1093/scan/nsaf114)
Supplement: nsaf114_Supplementary_Data [file nsaf114_supplementary_data.doc]

**Supplementary**

**S0: Unpacking interaction effects of reward and punishment on behavioural indices**

To further unpack an interaction effect of reward and threat on behavioural indices, we conducted separate repeated measures anova across each level of reward keeping punishment same, and each level of punishment keeping reward same (below table). Separate ANOVAs examining one factor while holding the other constant did not reveal a clearly identifiable source of the interactions.

| **Separate repeated measures anova on reaction time** | | | | |
| --- | --- | --- | --- | --- |
| **Reward Probability (fixed)** | **Main effect of punishment** | **Post-hoc comparison** | | |
|  |  | **Pair** | **Statistics** | |
| .25 | *F* (3, 117) = 1.64, *p* = .18, *η_p_^2^* = 0.04. | No comparison done | | |
| .50 | ‍*F* (3, 117) = 9.87, *p* < .001, *η_p_^2^* = 0.20.  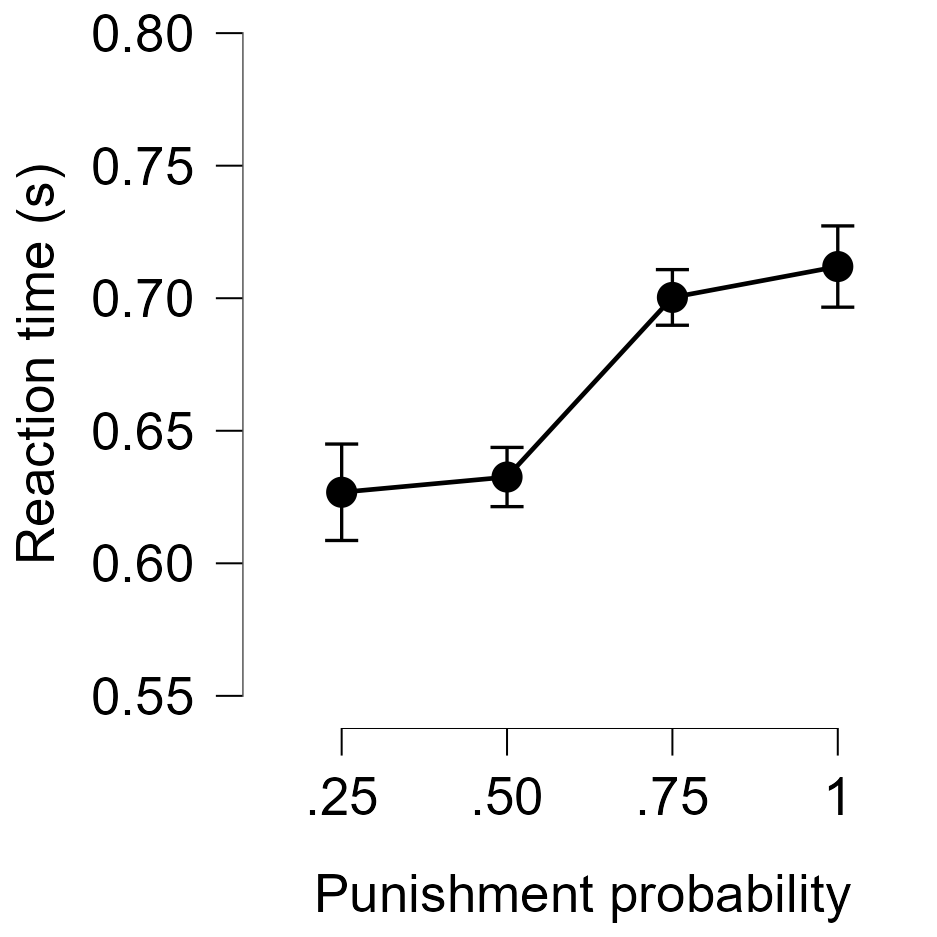 | .5–.25 vs .5–.5  .5–.25 vs .5–.75  .5–.25 vs .5–1  .5–.5 vs .5–.75  .5–.5 vs .5–1  .5–.75 vs .5–1 | *t*(39) = -0.33, *p* = .99, *d* = -0.04  *t*(39) = -3.15, *p* = .01, *d* = -0.58  *t*(39) = -3.09, *p* = .02, *d* = -0.67  *t*(39) = -4.21, *p* < .001, *d* = -0.53  *t*(39) = -3.86, *p* = .002, *d* = -0.62  *t*(39) = -1.03, *p* = .99, *d* = -0.09 | |
| .75 | ‍*F* (3, 117) = 18.02, *p* < .001, *η_p_^2^* = 0.31  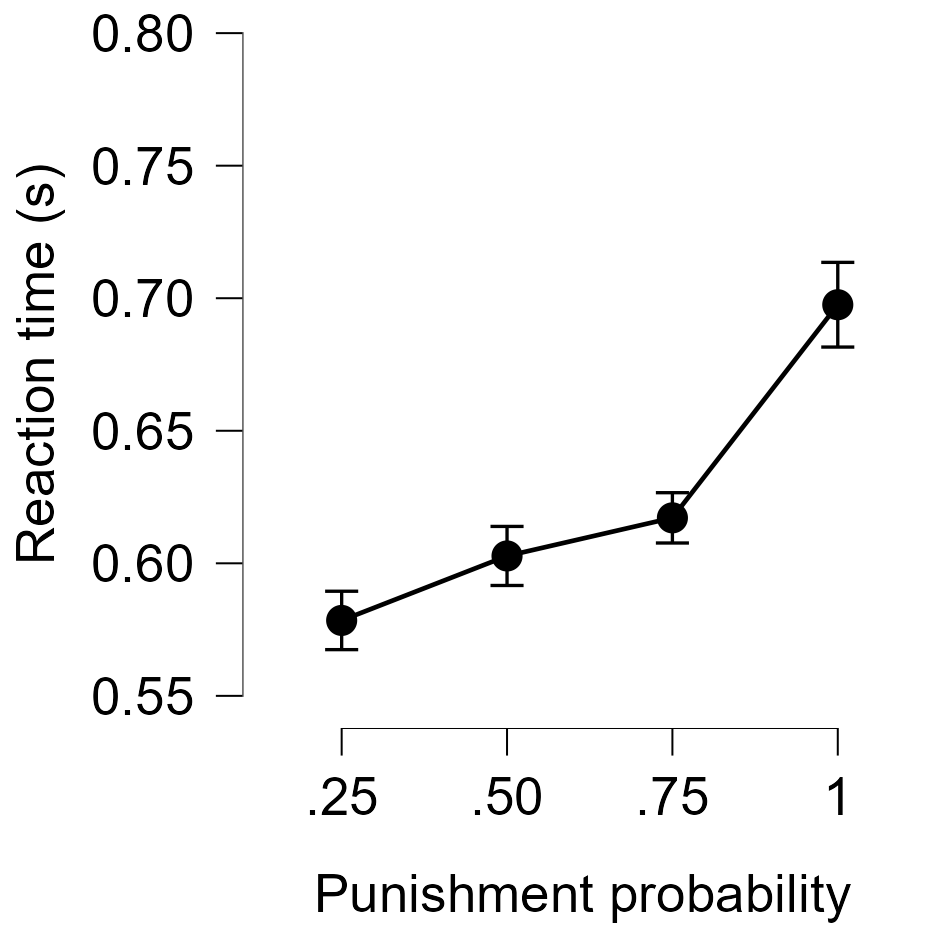 | .75–.25 vs .75–.5  .75–.25 vs .75–.75  .75–.25 vs .75–1  .75–.5 vs .75–.75  .75–.5 vs .75–1  .75–.75 vs .75–1 | *t*(39) = -2.71, *p* = .05, *d* = -0.20  *t*(39) = -2.38, *p* = .13, *d* = -0.32  *t*(39) = -5.53, *p* < .001, *d* = -0.98  *t*(39) = -0.97, *p* = .99, *d* = -0.11  *t*(39) = -4.17, *p* < .001, *d* = -0.78  *t*(39) = -5.30, *p* < .001, *d* = -0.66 | |
| 1 | ‍*F* (3, 117) = 5.39, *p* = .002, *η_p_^2^* = 0.12.+  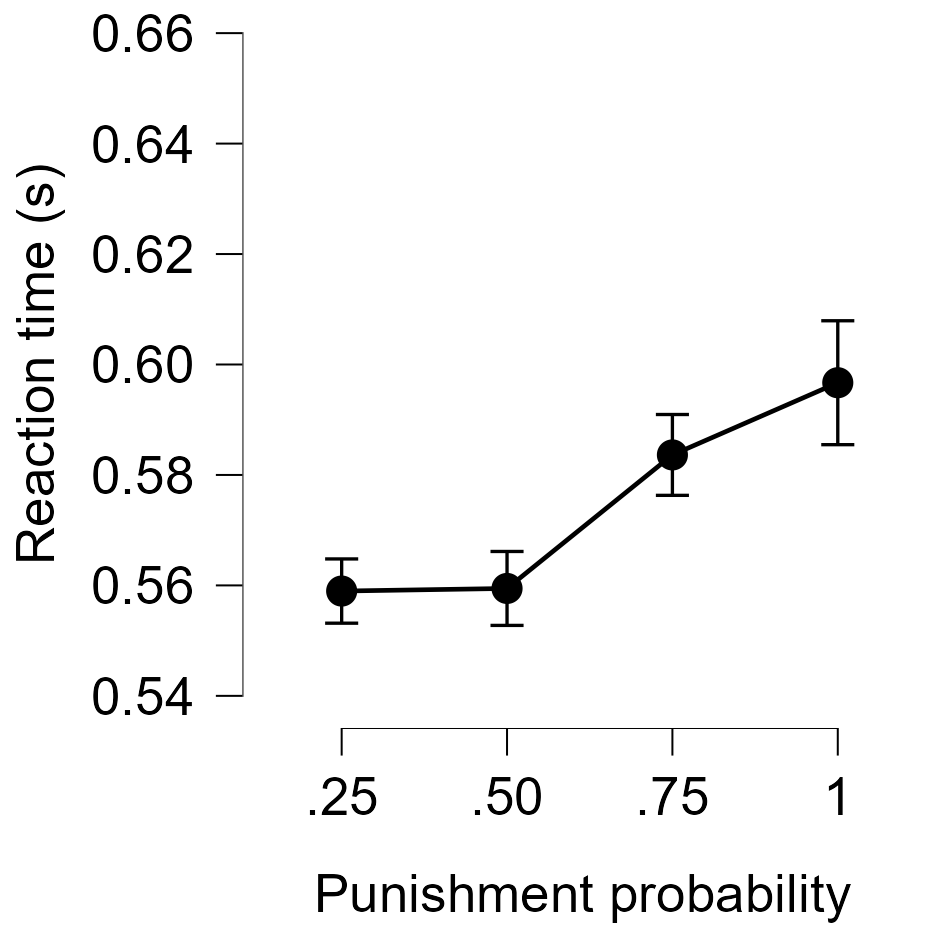 | 1–.25 vs 1–.5  1–.25 vs 1–.75  1–.25 vs 1–1  1–.5 vs 1–.75  1–.5 vs 1–1  1–.75 vs 1–1 | *t*(39) = -0.07, *p* = .99, *d* = -0.00  *t*(39) = -2.61, *p* = .07, *d* = -0.22  *t*(39) = -2.90, *p* = .03, *d* = -0.34  *t*(39) = -2.75, *p* = .05, *d* = -0.21  *t*(39) = -2.54, *p* = .09, *d* = -0.33  *t*(39) = -0.95, *p* = .99, *d* = -0.11 | |
| **Punishment Probability (fixed)** | **Main effect of reward** | **Post-hoc comparison** | | |
|  |  | **Pair** | | **Statistics** |
| .25 | *F* (3, 117) = 27.71, *p* < .001, *η_p_^2^* = 0.41.  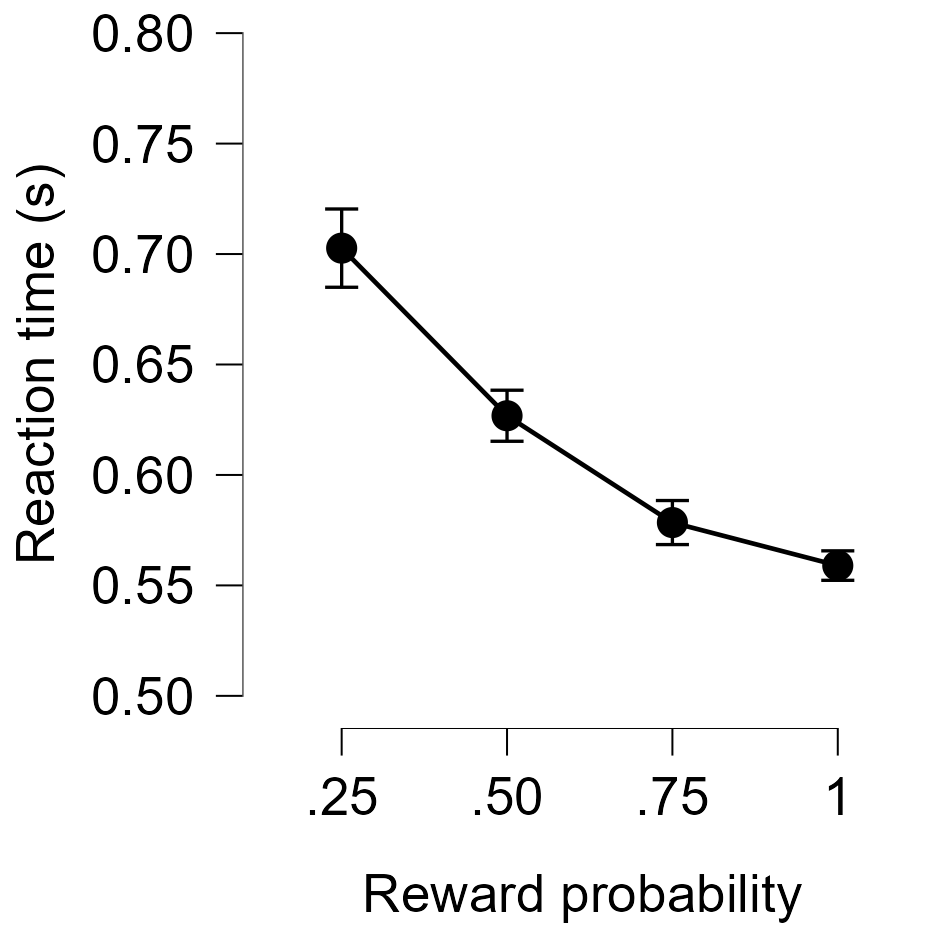 | .25–.25 vs .5–.25  .25–.25 vs .75–.25  .25–.25 vs 1–25  .5–.25 vs .75–.25  .5–.25 vs 1–.25  .75–.25 vs 1–.25 | *t*(39) = 3.31, *p* = .01, *d* = 0.65  *t*(39) = 5.51, *p* < .001, *d* = 1.07  *t*(39) = 7.45, *p* < .001, *d* = 1.24  *t*(39) = 3.57, *p* = .006, *d* = 0.42  *t*(39) = 5.45, *p* < .001, *d* = 0.58  *t*(39) = 2.72, *p* = .05, *d* = 0.16 | |
| .50 | ‍*F* (3, 117) = 29.67, *p* < .001, *η_p_^2^* = 0.43.  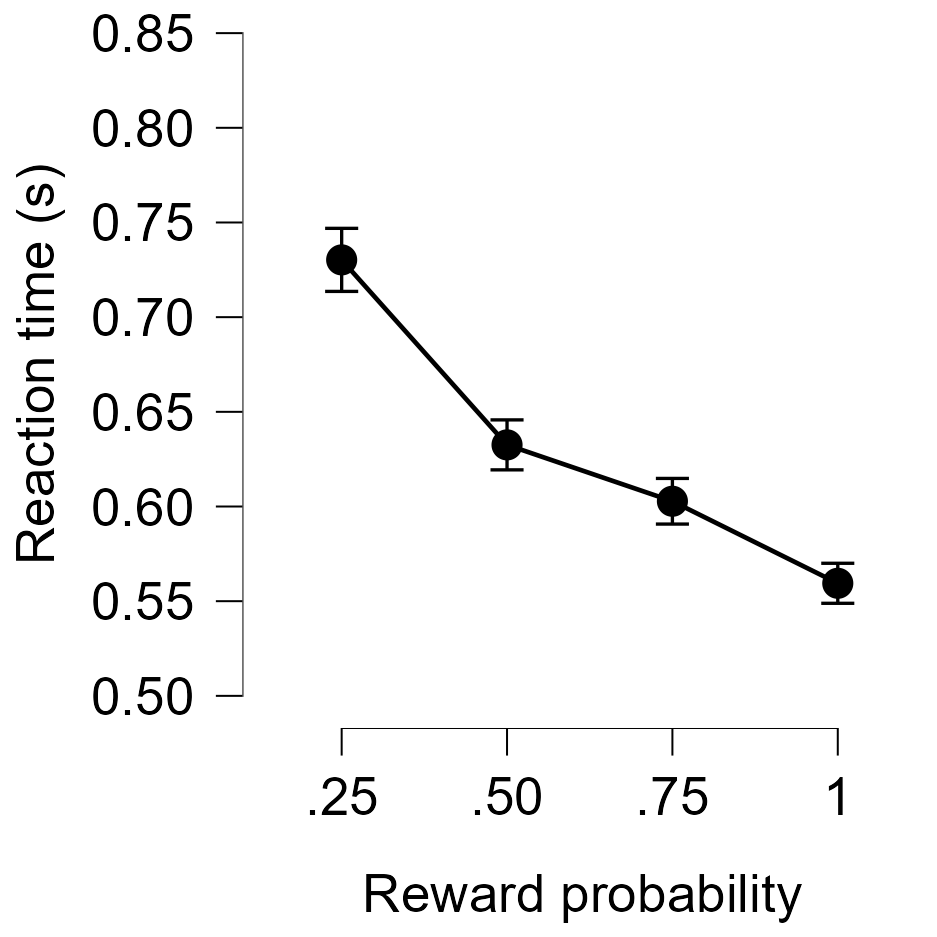 | .25–.5 vs .5–.5  .25–.5 vs .75–.5  .25–.5 vs 1–5  .5–.5 vs .75–.5  .5–.5 vs 1–.5  .75–.5 vs 1–.5 | *t*(39) = 5.17, *p* < .001, *d* = 0.82  *t*(39) = 5.39, *p* < .001, *d* = 1.07  *t*(39) = 8.05, *p* < .001, *d* = 1.44  *t*(39) = 1.60, *p* = .70, *d* = 0.25  *t*(39) = 3.89, *p* = .002, *d* = 0.61  *t*(39) = 5.38, *p* < .001, *d* = 0.36 | |
| .75 | ‍*F* (3, 117) = 21.81, *p* < .001, *η_p_^2^* = 0.35  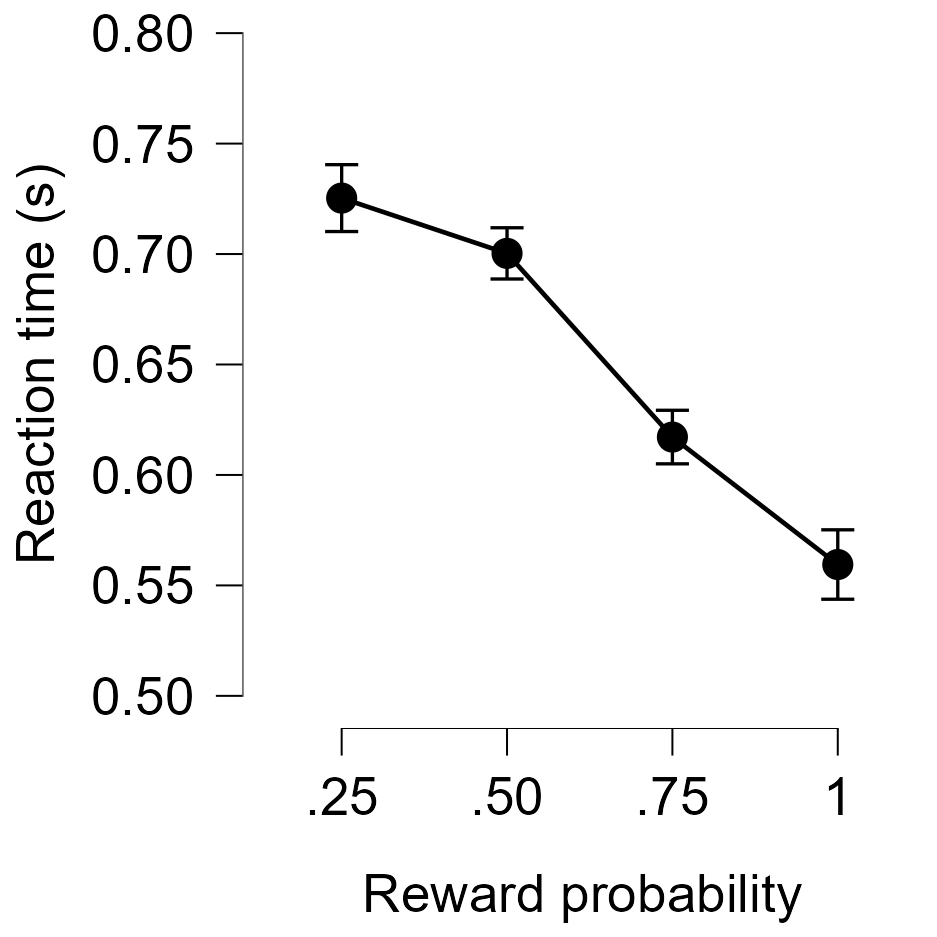 | .25–.75 vs .5–.75  .25–.75 vs .75–.75  .25–.75 vs 1–.75  .5–.75 vs .75–.75  .5–.75 vs 1–.75  .75–.75 vs 1–.75 | *t*(39) = 1.76, *p* = .051, *d* = 0.20  *t*(39) = 4.94, *p* < .001, *d* = 0.89  *t*(39) = 5.30, *p* < .001, *d* = 1.17  *t*(39) = 4.93, *p* < .001, *d* = 0.68  *t*(39) = 4.82, *p* < .001, *d* = 0.96  *t*(39) = 2.28, *p* = .16, *d* = 0.27 | |
| 1 | ‍*F* (3, 117) = 22.03, *p* < .001, *η_p_^2^* = 0.36.  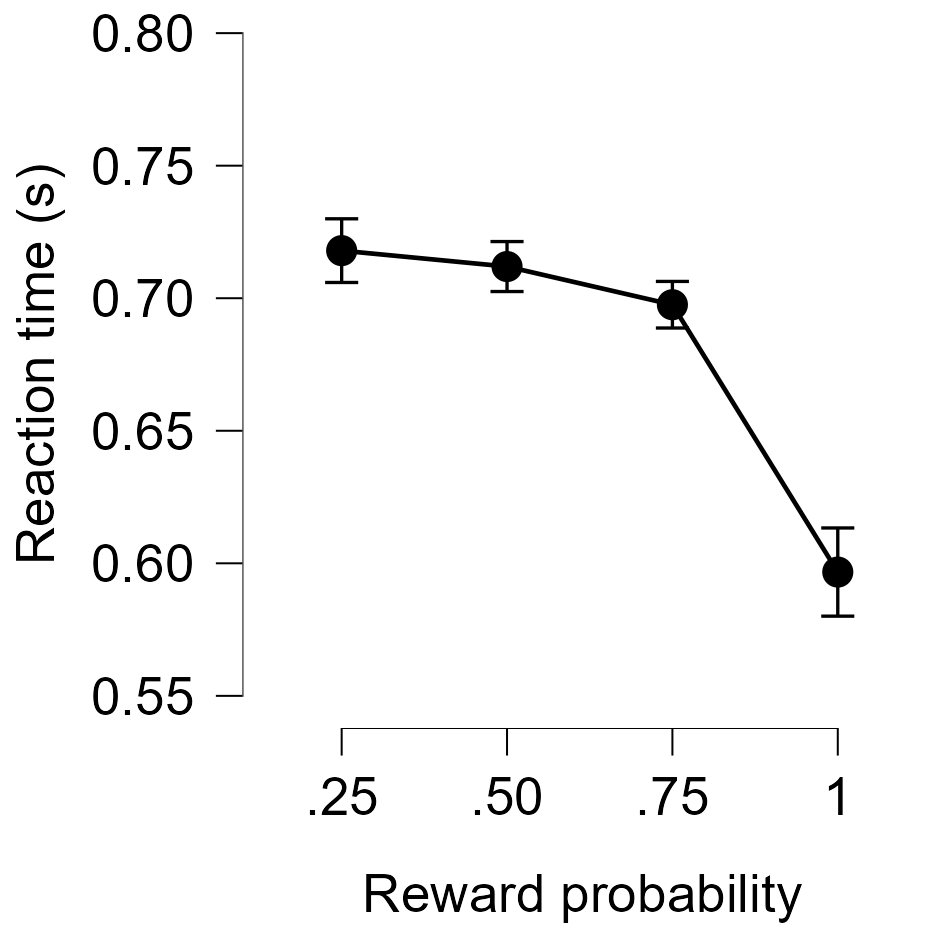 | .25–1 vs .5–1  .25–1 vs .75–1  .25–1 vs 1–1  .5–1 vs .75–1  .5–1 vs 1–1  .75–1 vs 1–1 | *t*(39) = 0.59, *p* = .99, *d* = 0.04  *t*(39) = 1.31, *p* < .99, *d* = 0.16  *t*(39) = 5.26, *p* < .001, *d* = 0.98  *t*(39) = 1.18, *p* < .99, *d* = 0.11  *t*(39) = 5.39, *p* < .001, *d* = 0.93  t(39) = 5.99, p < .001, d = 0.81 | |

| **Separate repeated measures anova on rejection rate** | | | |
| --- | --- | --- | --- |
| **Reward Probability (fixed)** | **Main effect of punishment** | **Post-hoc comparison** | |
|  |  | **Pair** | **Statistics** |
| .25 | *F* (3, 117) = 41.05, *p* < .001, *η_p_^2^* = 0.51.  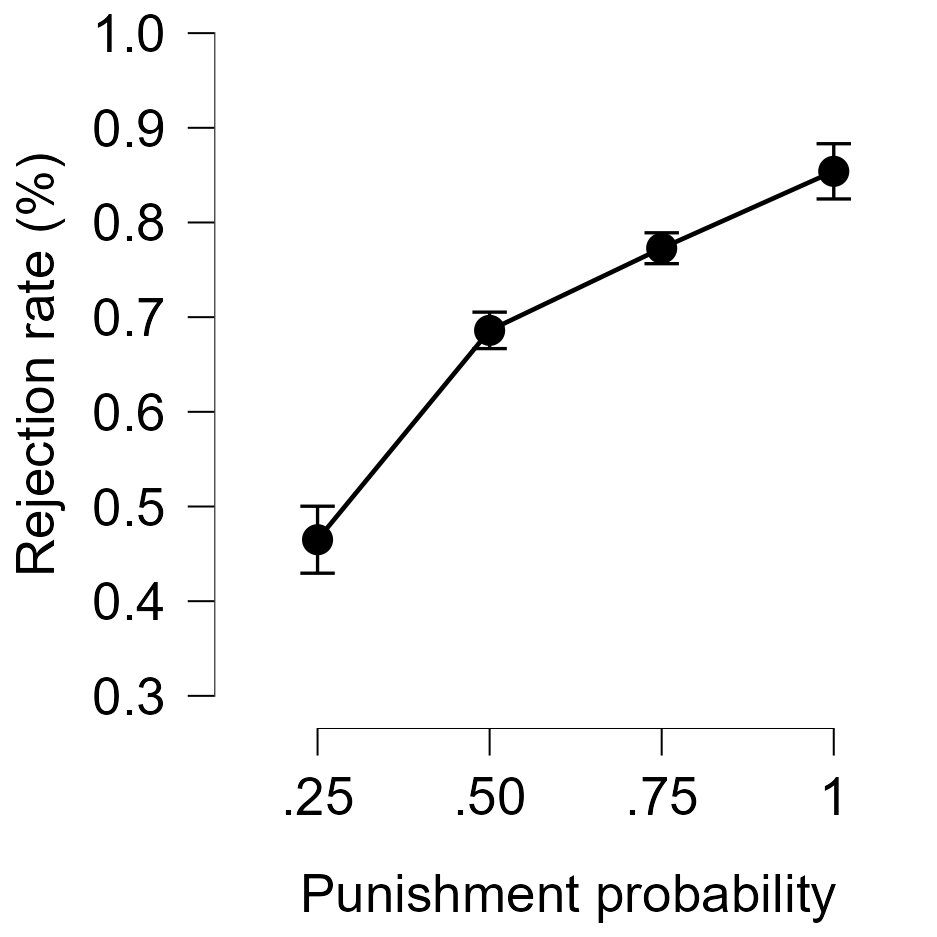 | .25–.25 vs .25–.5  .25–.25 vs .25–.75  .25–.25 vs .25–1  .25–.5 vs .25–.75  .25–.5 vs .25–1  .25–.75 vs .25–1 | *t*(39) = -5.85, *p* < .001, *d* = -0.67  *t*(39) = -7.21, *p* < .001, *d* = -0.94  *t*(39) = -7.69, *p* < .001, *d* = -1.19  *t*(39) = -4.56, *p* < .001, *d* = -0.26  *t*(39) = -4.52, *p* < .001, *d* = -0.51  *t*(39) = -3.13, *p* = .08, *d* = -0.24 |
| .50 | ‍*F* (3, 117) = 60.46, *p* < .001, *η_p_^2^* = 0.60.  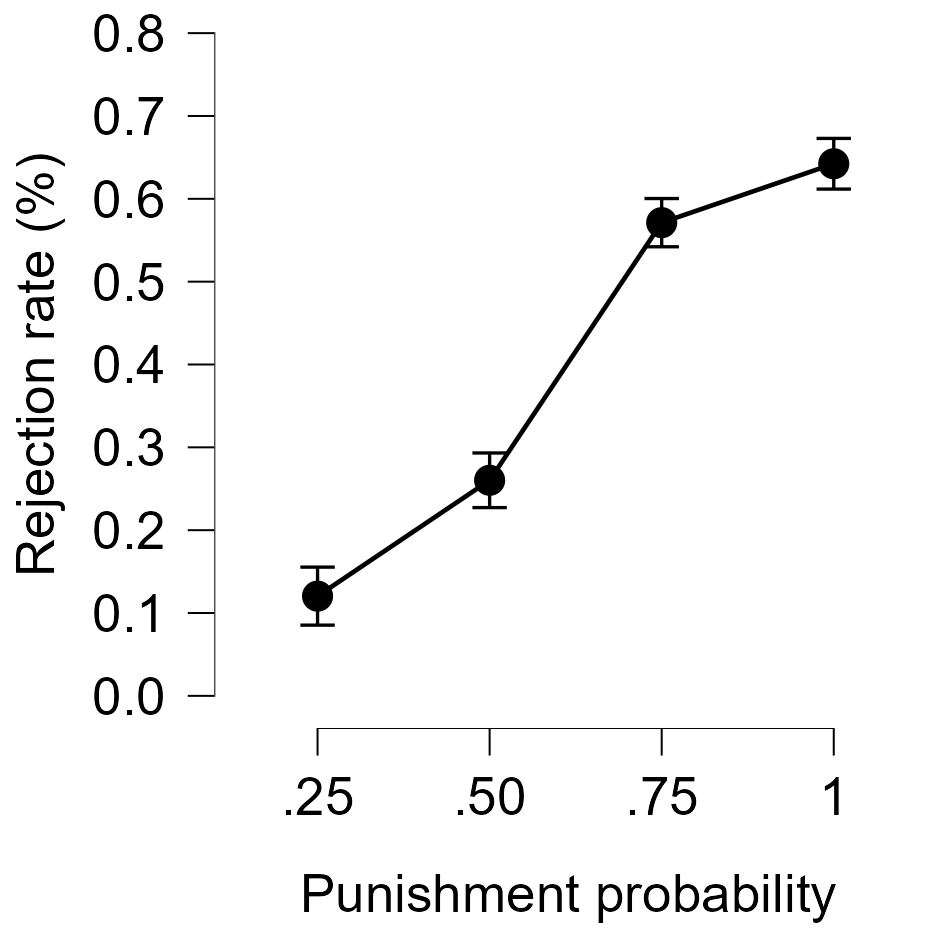 | .5–.25 vs .5–.5  .5–.25 vs .5–.75  .5–.25 vs .5–1  .5–.5 vs .5–.75  .5–.5 vs .5–1  .5–.75 vs .5–1 | *t*(39) = -4.41, *p* = .001, *d* = -0.40  *t*(39) = -8.44, *p* < .001, *d* = -1.29  *t*(39) = -9.92, *p* < .001, *d* = -1.49  *t*(39) = -6.31, *p* < .001, *d* = -0.89  *t*(39) = -7.25, *p* < .001, *d* = -1.09  *t*(39) = -3.85, *p* = .003, *d* = -0.20 |
| .75 | ‍*F* (3, 117) = 38.10, *p* < .001, *η_p_^2^* = 0.49  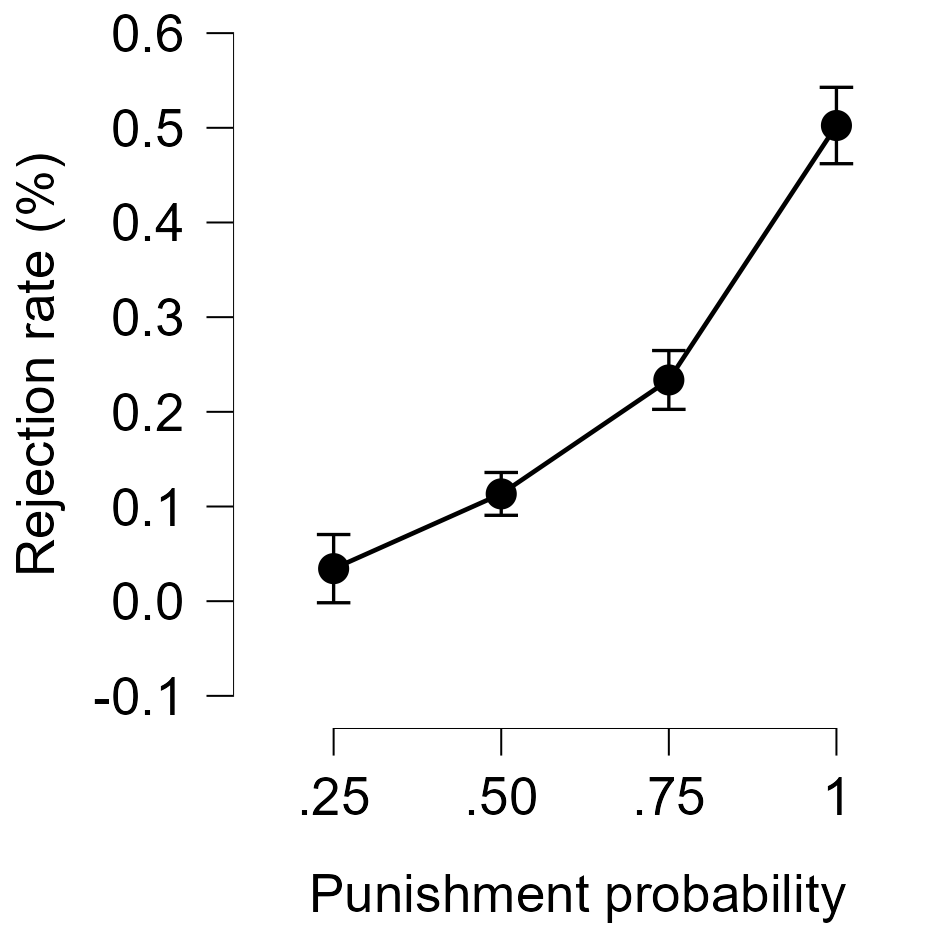 | .75–.25 vs .75–.5  .75–.25 vs .75–.75  .75–.25 vs .75–1  .75–.5 vs .75–.75  .75–.5 vs .75–1  .75–.75 vs .75–1 | *t*(39) = -2.65, *p* = .069, *d* = -0.25  *t*(39) = -3.70, *p* = .004, *d* = -0.64  *t*(39) = -8.01, *p* < .001, *d* = -1.52  *t*(39) = -3.42, *p* = .009, *d* = -0.39  *t*(39) = -7.47, *p* < .001, *d* = -1.26  *t*(39) = -5.92, *p* < .001, *d* = -0.87 |
| 1 | ‍*F* (3, 117) = 13.32, *p* < .001, *η_p_^2^* = 0.25.  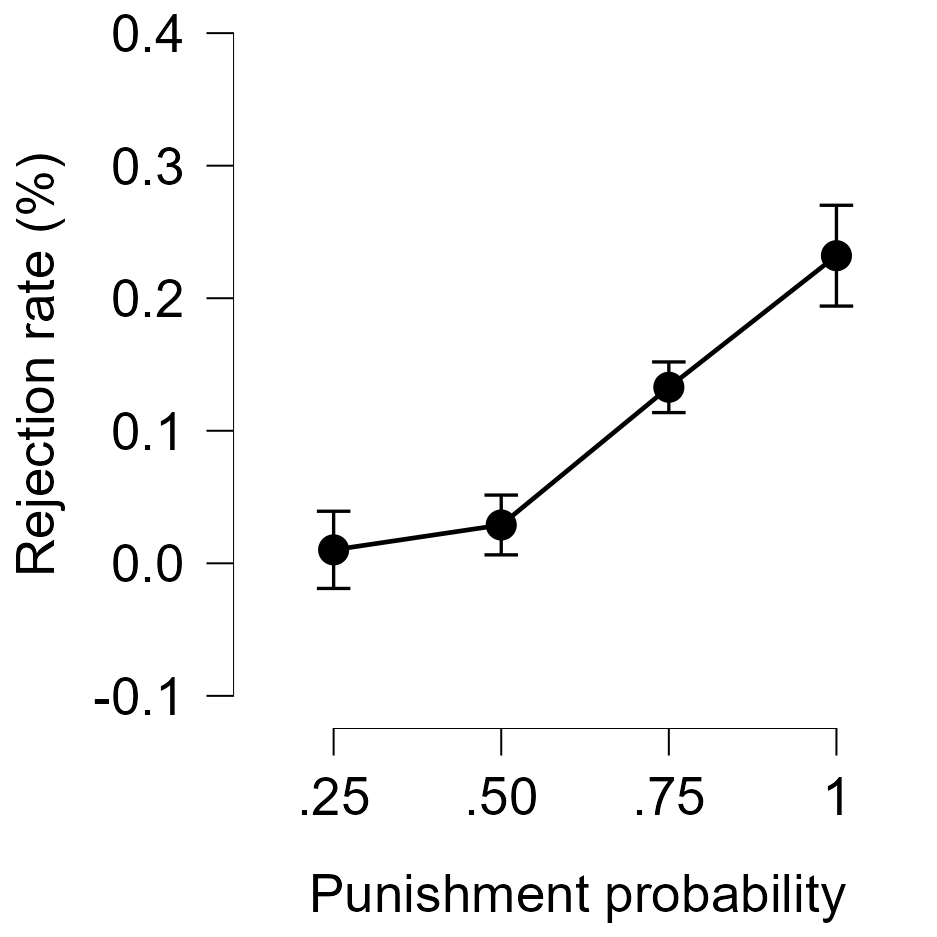 | 1–.25 vs 1–.5  1–.25 vs 1–.75  1–.25 vs 1–1  1–.5 vs 1–.75  1–.5 vs 1–1  1–.75 vs 1–1 | *t*(39) = -1.13, *p* = .99, *d* = -0.08  *t*(39) = -3.11, *p* = .021, *d* = -0.52  *t*(39) = -3.94, *p* = .002, *d* = -0.94  *t*(39) = -3.23, *p* = .015, *d* = -0.44  *t*(39) = -3.93, *p* = .002, *d* = -0.86  *t*(39) = -3.33, *p* = .011, *d* = -0.42 |
| **Punishment Probability (fixed)** | **Main effect of reward** | **Post-hoc comparison** | |
|  |  | **Pair** | **Statistics** |
| .25 | *F* (3, 117) = 46.04, *p* < .001, *η_p_^2^* = 0.54.  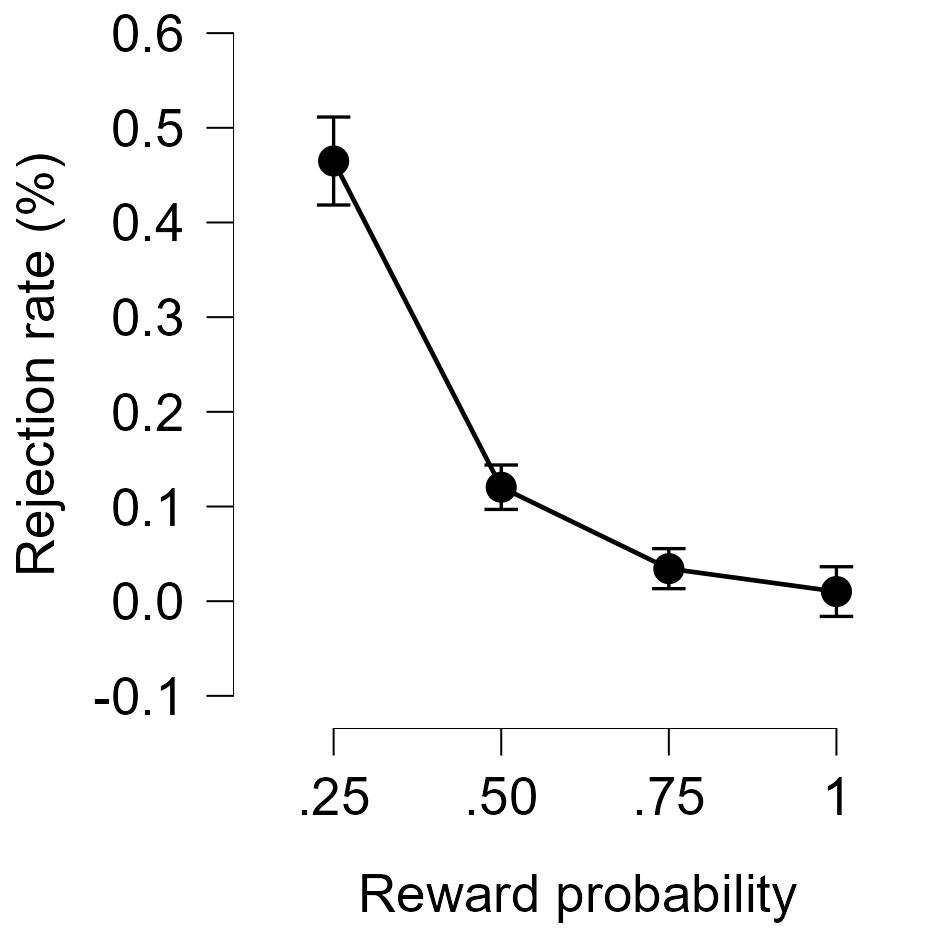 | .25–.25 vs .5–.25  .25–.25 vs .75–.25  .25–.25 vs 1–25  .5–.25 vs .75–.25  .5–.25 vs 1–.25  .75–.25 vs 1–.25 | *t*(39) = 6.84, *p* = .01, *d* = 1.47  *t*(39) = 7.50, *p* < .001, *d* = 1.83  *t*(39) = 7.65, *p* < .001, *d* = 1.94  *t*(39) = 3.12, *p* = .02, *d* = 0.36  *t*(39) = 3.12, *p* = .02, *d* = 0.47  *t*(39) = 1.82, *p* = .10, *d* = 0.10 |
| .50 | ‍*F* (3, 117) = 72.95, *p* < .001, *η_p_^2^* = 0.65.  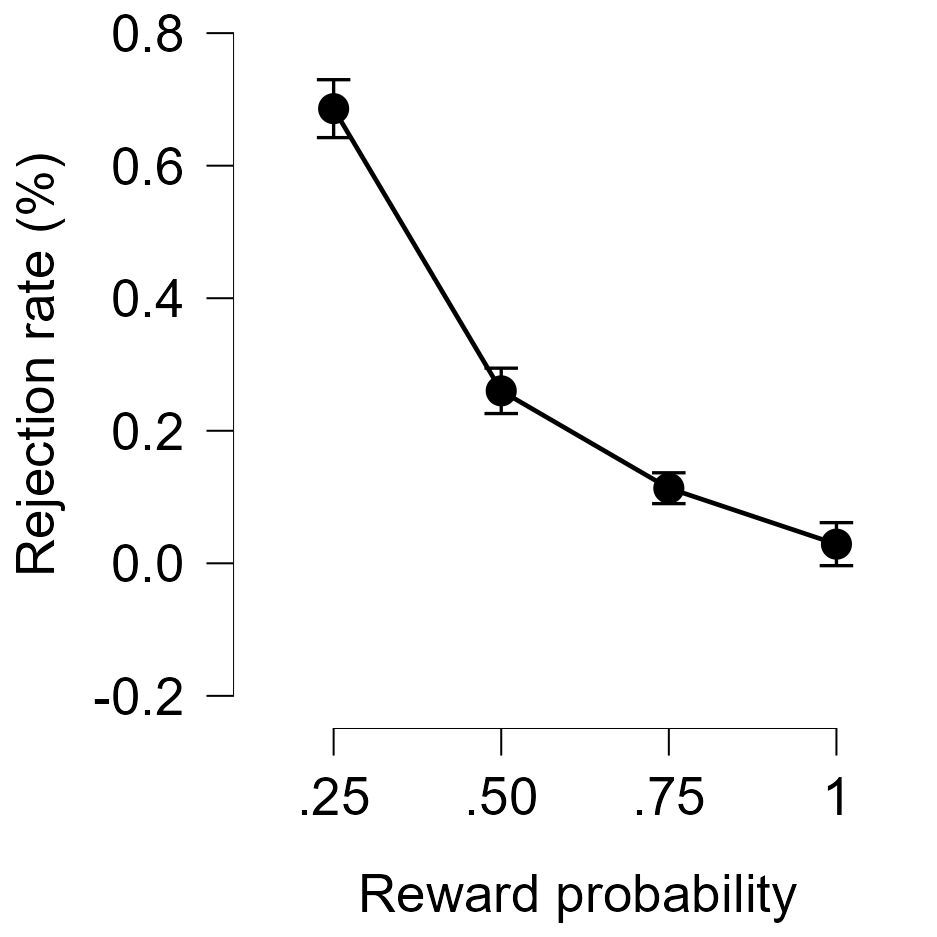 | .25–.5 vs .5–.5  .25–.5 vs .75–.5  .25–.5 vs 1–5  .5–.5 vs .75–.5  .5–.5 vs 1–.5  .75–.5 vs 1–.5 | *t*(39) = 7.68, *p* < .001, *d* = 1.45  *t*(39) = 10.31, *p* < .001, *d* = 1.95  *t*(39) = 11.83, *p* < .001, *d* = 2.24  *t*(39) = 4.22, *p* < .001, *d* = 0.50  *t*(39) = 4.41, *p* < .001, *d* = 0.78  *t*(39) = 2.95, *p* = .03, *d* = 0.28 |
| .75 | ‍*F* (3, 117) = 74.11, *p* < .001, *η_p_^2^* = 0.65  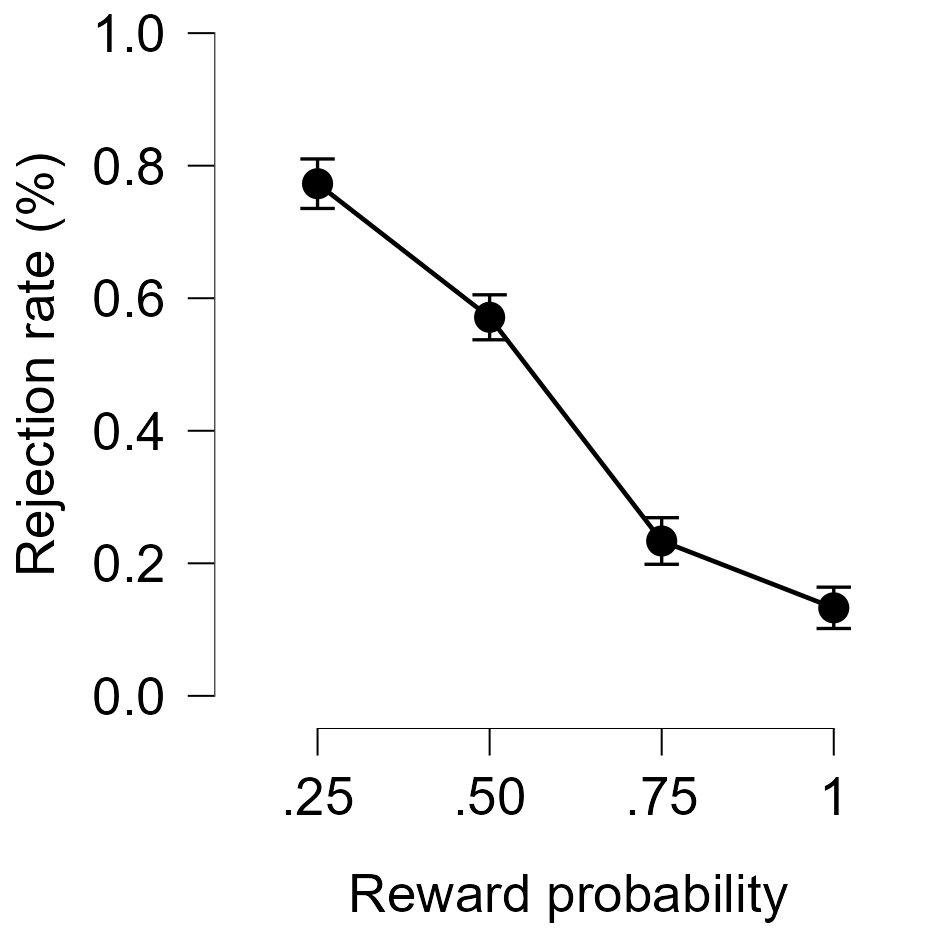 | .25–.75 vs .5–.75  .25–.75 vs .75–.75  .25–.75 vs 1–.75  .5–.75 vs .75–.75  .5–.75 vs 1–.75  .75–.75 vs 1–.75 | *t*(39) = 5.34, *p* < .001, *d* = 0.59  *t*(39) = 9.00, *p* < .001, *d* = 1.58  *t*(39) = 12.22, *p* < .001, *d* = 1.87  *t*(39) = 6.41, *p* < .001, *d* = 0.98  *t*(39) = 8.24, *p* < .001, *d* = 1.28  *t*(39) = 3.33, *p* = .011, *d* = 0.29 |
| 1 | ‍*F* (3, 117) = 58.82, *p* < .001, *η_p_^2^* = 0.60.  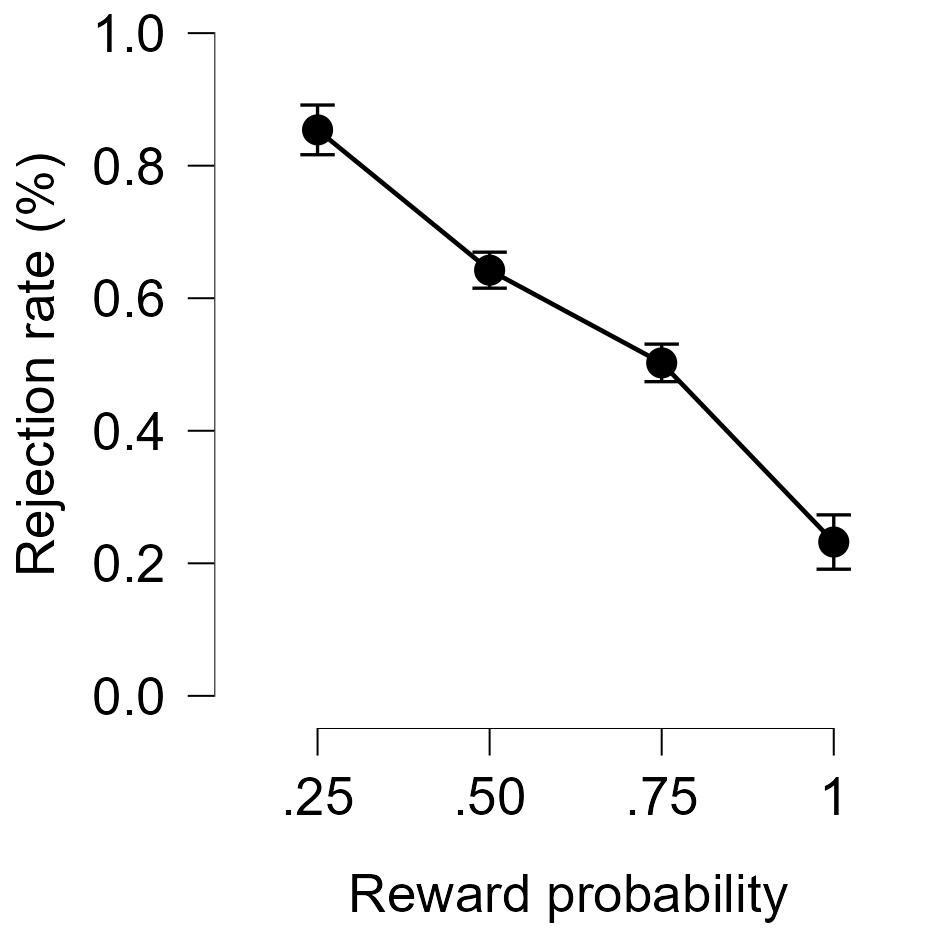 | .25–1 vs .5–1  .25–1 vs .75–1  .25–1 vs 1–1  .5–1 vs .75–1  .5–1 vs 1–1  .75–1 vs 1–1 | *t*(39) = 5.22, *p* < .001, *d* = 0.61  *t*(39) = 6.71, *p* < .001, *d* = 1.01  *t*(39) = 10.82, *p* < .001, *d* = 1.79  *t*(39) = 4.46, *p* < .001, *d* = 0.40  *t*(39) = 7.42, *p* < .001, *d* = 1.18  *t*(39) = 5.82, *p* < .001, *d* = 0.78 |

| **Separate repeated measures anova on BCI** | | | |
| --- | --- | --- | --- |
| **Reward Probability (fixed)** | **Main effect of punishment** | **Post-hoc comparison** | |
|  |  | **Pair** | **Statistics** |
| .25 | *F* (3, 117) = 0.28, *p* = .83, *η_p_^2^* = 0.007. | No comparison done | |
| .50 | ‍*F* (3, 117) = 5.97, *p* < .001, *η_p_^2^* = 0.13.  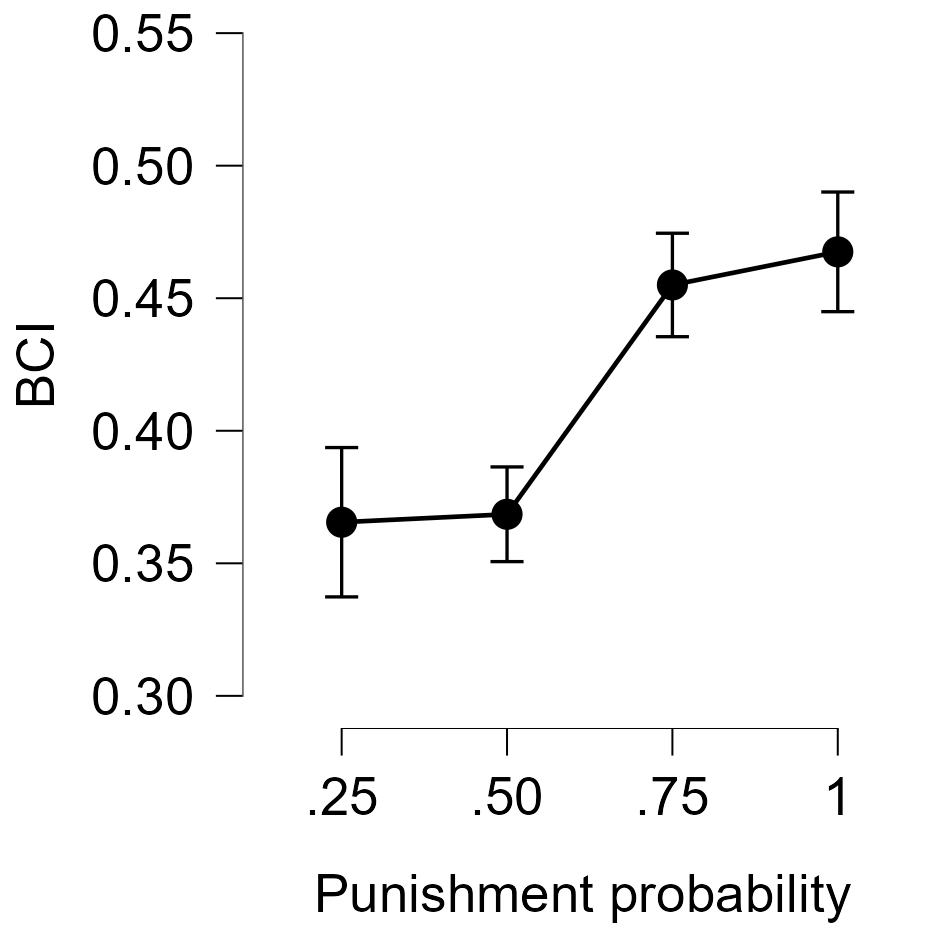 | .5–.25 vs .5–.5  .5–.25 vs .5–.75  .5–.25 vs .5–1  .5–.5 vs .5–.75  .5–.5 vs .5–1  .5–.75 vs .5–1 | *t*(39) = -0.11, *p* = .999, *d* = -0.02  *t*(39) = -2.28, *p* < .167, *d* = -0.58  *t*(39) = -2.50, *p* < .100, *d* = -0.66  *t*(39) = -3.14, *p* < .019, *d* = -0.56  *t*(39) = -3.10, *p* = .021, *d* = -0.64  *t*(39) = -0.66, *p* = .999, *d* = -0.08 |
| .75 | ‍*F* (3, 117) = 10.14, *p* < .001, *η_p_^2^* = 0.20  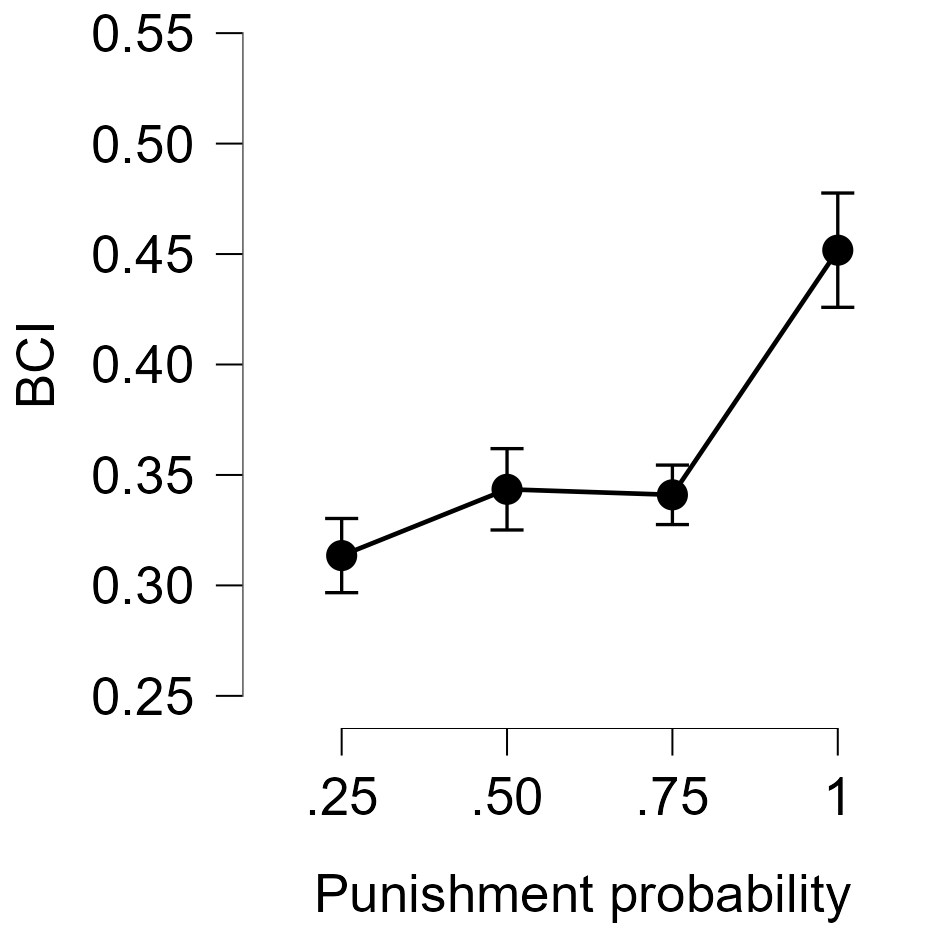 | .75–.25 vs .75–.5  .75–.25 vs .75–.75  .75–.25 vs .75–1  .75–.5 vs .75–.75  .75–.5 vs .75–1  .75–.75 vs .75–1 | *t*(39) = -1.53, *p* = .802, *d* = -0.22  *t*(39) = -1.26, *p* = .999, *d* = -0.20  *t*(39) = -4.18, *p* < .001, *d* = -1.05  *t*(39) = -0.11, *p* = .999, *d* = -0.01  *t*(39) = -3.01, *p* < .027, *d* = -0.82  *t*(39) = -4.10, *p* = .001, *d* = -0.84 |
| 1 | ‍*F* (3, 117) = 4.42, *p* = .005, *η_p_^2^* = 0.10.  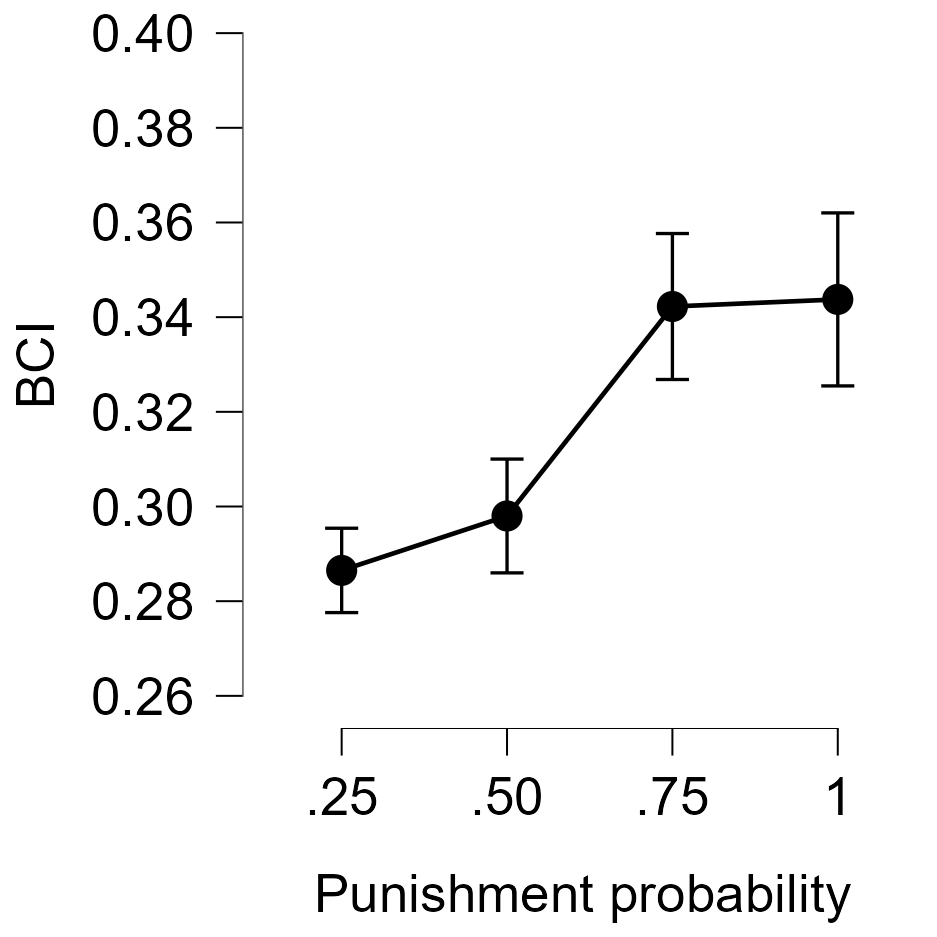 | 1–.25 vs 1–.5  1–.25 vs 1–.75  1–.25 vs 1–1  1–.5 vs 1–.75  1–.5 vs 1–1  1–.75 vs 1–1 | *t*(39) = -1.25, *p* = .999, *d* = -0.09  *t*(39) = -3.08, *p* = .023, *d* = -0.46  *t*(39) = -2.78, *p* = .050, *d* = -0.47  *t*(39) = -2.27, *p* = .170, *d* = -0.36  *t*(39) = -1.91, *p* = .375, *d* = -0.38  *t*(39) = -0.06, *p* = .999, *d* = -0.01 |
| **Punishment Probability (fixed)** | **Main effect of reward** | **Post-hoc comparison** | |
|  |  | **Pair** | **Statistics** |
| .25 | *F* (3, 117) = 18.92, *p* < .001, *η_p_^2^* = 0.32.  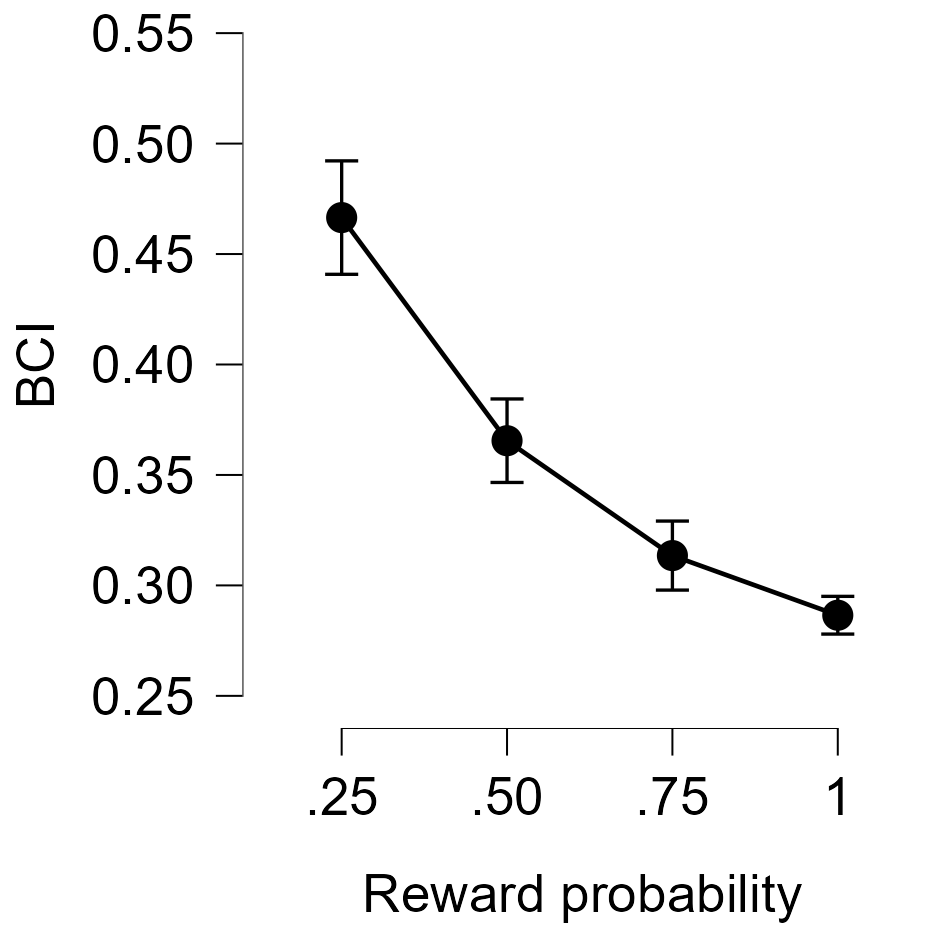 | .25–.25 vs .5–.25  .25–.25 vs .75–.25  .25–.25 vs 1–25  .5–.25 vs .75–.25  .5–.25 vs 1–.25  .75–.25 vs 1–.25 | *t*(39) = 2.92, *p* = .035, *d* = 0.78  *t*(39) = 4.69, *p* < .001, *d* = 1.19  *t*(39) = 6.70, *p* < .001, *d* = 1.40  *t*(39) = 2.26, *p* = .173, *d* = 0.40  *t*(39) = 4.21, *p* < .001, *d* = 0.61  *t*(39) = 2.23, *p* = .187, *d* = 0.21 |
| .50 | ‍*F* (3, 117) = 12.31, *p* < .001, *η_p_^2^* = 0.24.  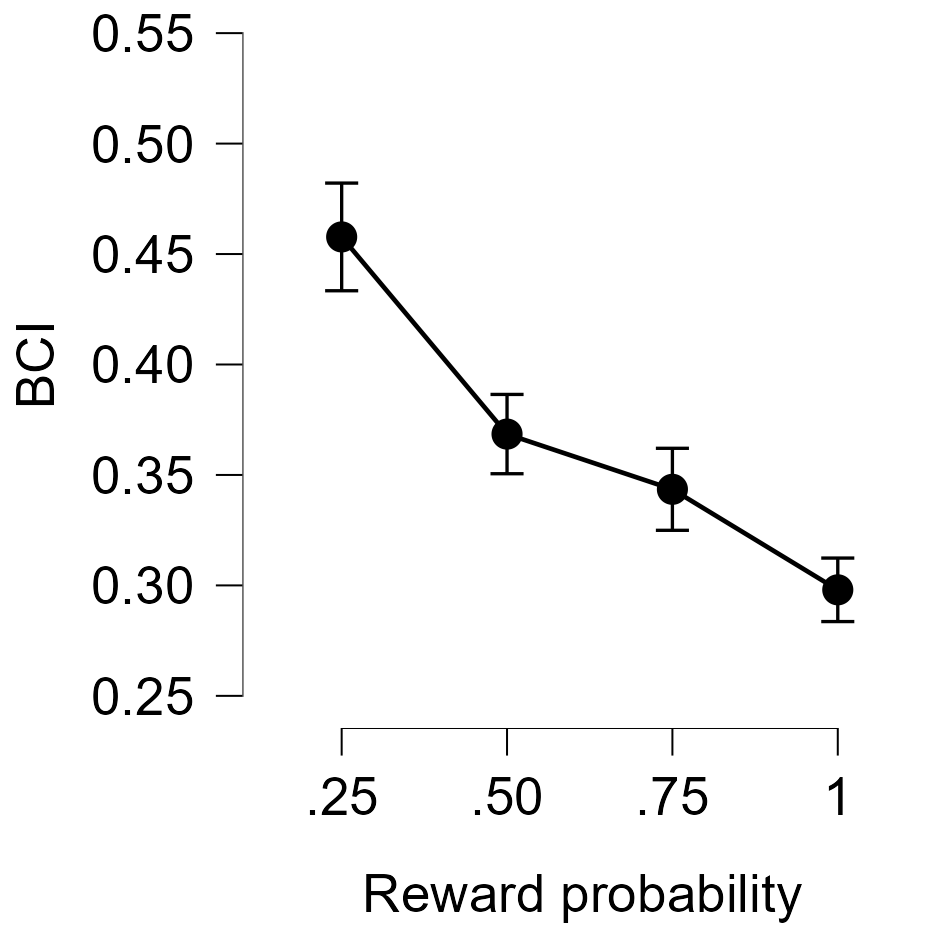 | .25–.5 vs .5–.5  .25–.5 vs .75–.5  .25–.5 vs 1–5  .5–.5 vs .75–.5  .5–.5 vs 1–.5  .75–.5 vs 1–.5 | *t*(39) = 3.06, *p* = .024, *d* = 0.70  *t*(39) = 3.30, *p* < .012, *d* = 0.90  *t*(39) = 5.51, *p* < .001, *d* = 1.26  *t*(39) = 0.98, *p* < .999, *d* = 0.19  *t*(39) = 2.93, *p* = .034, *d* = 0.55  *t*(39) = 2.62, *p* = .075, *d* = 0.36 |
| .75 | ‍*F* (3, 117) = 8.18, *p* < .001, *η_p_^2^* = 0.17  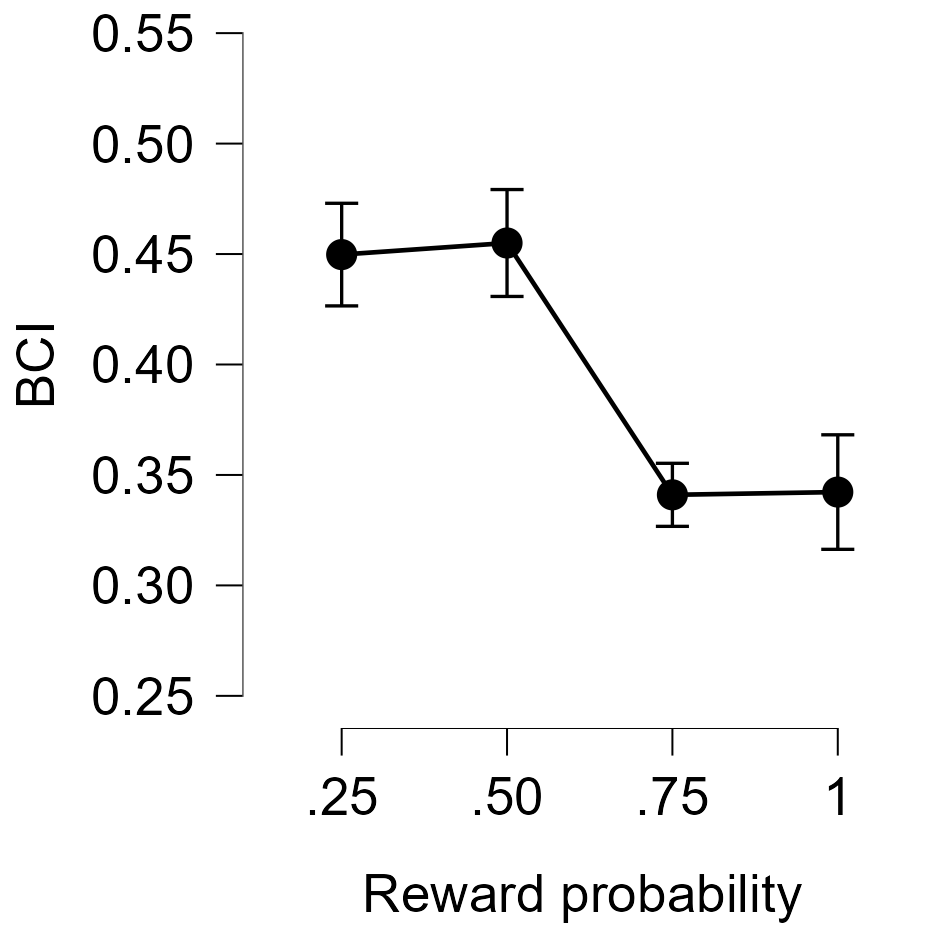 | .25–.75 vs .5–.75  .25–.75 vs .75–.75  .25–.75 vs 1–.75  .5–.75 vs .75–.75  .5–.75 vs 1–.75  .75–.75 vs 1–.75 | *t*(39) = -0.17, *p* = .999, *d* = -0.03  *t*(39) = 3.74, *p* = .004, *d* = 0.75  *t*(39) = 2.85, *p* = .041, *d* = 0.75  *t*(39) = 4.07, *p* = .001, *d* = 0.79  *t*(39) = 2.80, *p* = .047, *d* = 0.78  *t*(39) = -0.05, *p* = .999, *d* = -0.00 |
| 1 | ‍*F* (3, 117) = 6.71, *p* < .001, *η_p_^2^* = 0.14.  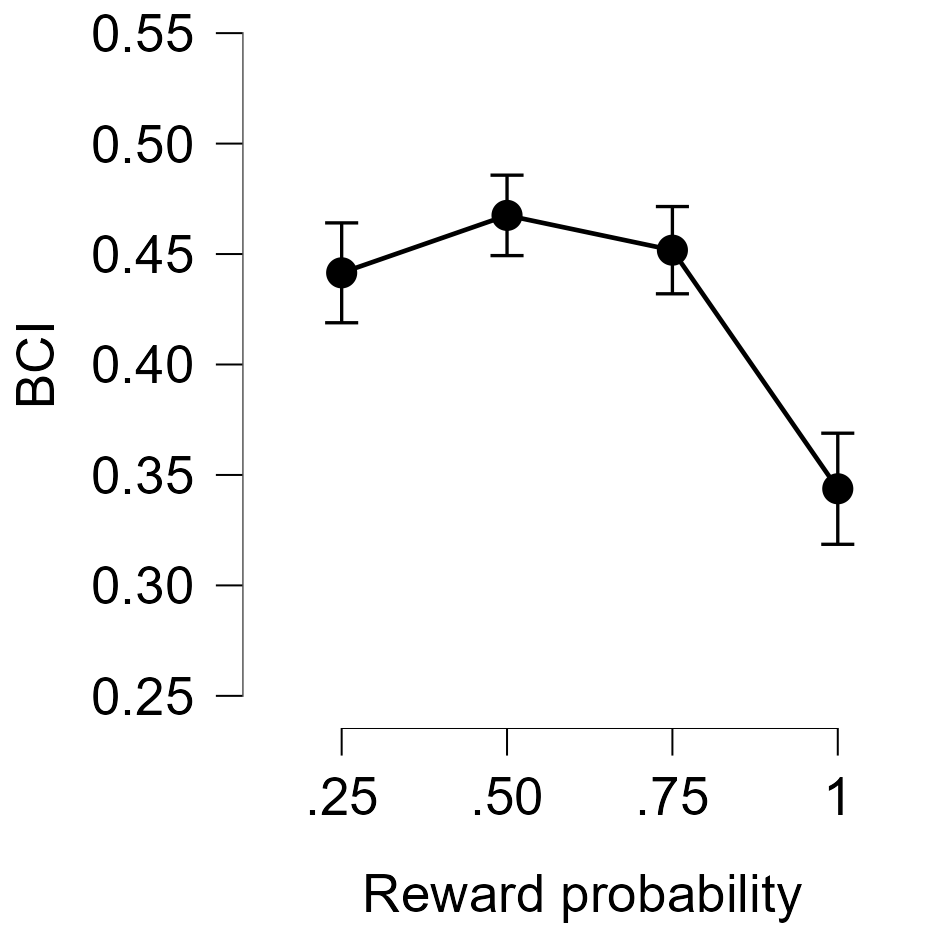 | .25–1 vs .5–1  .25–1 vs .75–1  .25–1 vs 1–1  .5–1 vs .75–1  .5–1 vs 1–1  .75–1 vs 1–1 | *t*(39) = -1.05, *p* = .999, *d* = -0.16  *t*(39) = -0.31, *p* = .999, *d* = -0.06  *t*(39) = 2.74, *p* = .055, *d* = 0.61  *t*(39) = 0.62, *p* = .999, *d* = 0.09  *t*(39) = 3.65, *p* = .005, *d* = 0.77  *t*(39) = 3.63, *p* = .005, *d* = 0.67 |

1. **S1. Line plots for behavioural measures**
2.
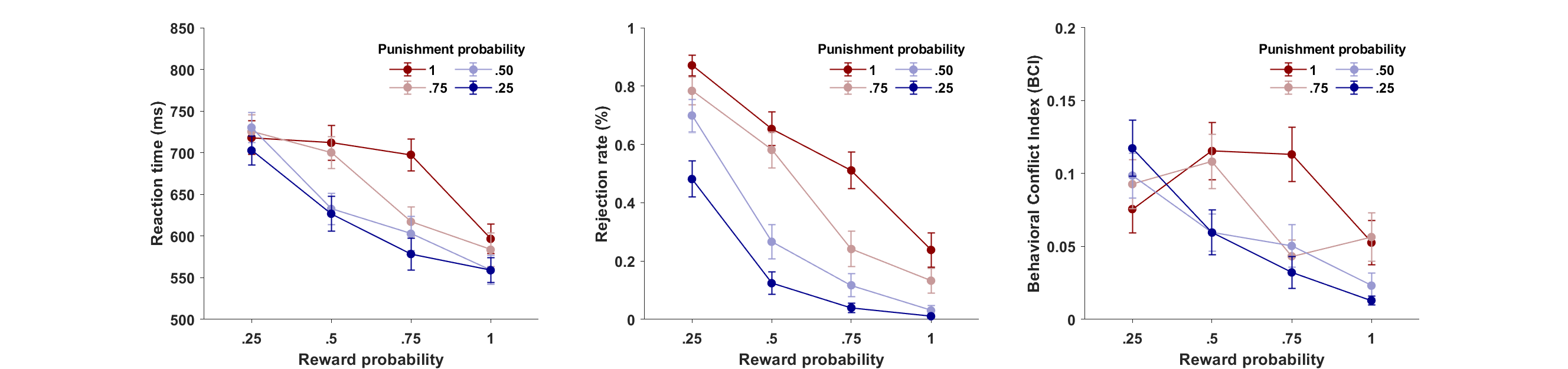

3. Line plot for objective MFT power for 16 conditions
4.
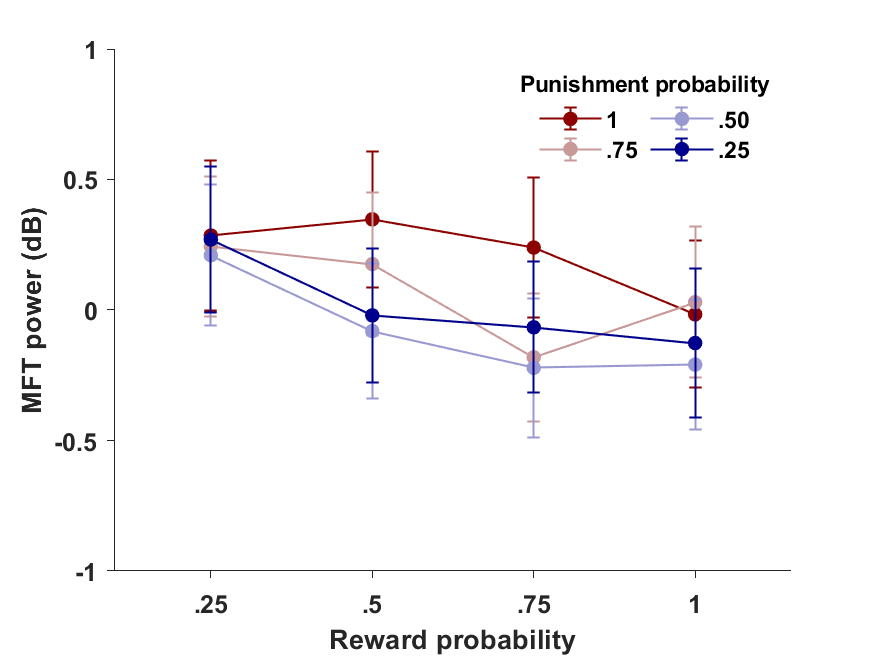

5. **S2. Individualised conflict conditions from 1^st^ (maximum) to 16^th^ (minimum) conflict for each participant based on BCI score**

|  | 1. **Ordered conflict condition (1^st^ is maximum conflict, 16^th^ is minimum conflict)** | | | | | | | | | | | | | | | |
| --- | --- | --- | --- | --- | --- | --- | --- | --- | --- | --- | --- | --- | --- | --- | --- | --- |
| 1. **Participant** | 1. **1^st^** | 1. **2^nd^** | 1. **3^rd^** | 1. **4^th^** | 1. **5^th^** | 1. **6^th^** | 1. **7^th^** | 1. **8^th^** | 1. **9^th^** | 1. **10^th^** | 1. **11^th^** | 1. **12^th^** | 1. **13^th^** | 1. **14^th^** | 1. **15^th^** | 1. **16^th^** |
| 1. 1 | 1. 1-.75 | 1. .5-.25 | 1. .75-.5 | 1. .75-.75 | 1. .75-.25 | 1. .25-.25 | 1. .25-.5 | 1. .5-.5 | 1. .5-.75 | 1. .75-1 | 1. 1-.5 | 1. 1-1 | 1. .25-.75 | 1. .25-1 | 1. .5-1 | 1. 1-.25 |
| 1. 2 | 1. .75-1 | 1. .25-.5 | 1. .5-.75 | 1. .5-1 | 1. .25-.75 | 1. 1-1 | 1. .5-.25 | 1. .25-.25 | 1. .75-.25 | 1. .25-1 | 1. .5-.5 | 1. .75-.5 | 1. .75-.75 | 1. 1-.25 | 1. 1-.5 | 1. 1-.75 |
| 1. 3 | 1. .25-.75 | 1. .25-1 | 1. .5-1 | 1. .25-.5 | 1. .25-.25 | 1. .75-1 | 1. .5-.75 | 1. .75-.75 | 1. .5-.25 | 1. .5-.5 | 1. .75-.25 | 1. .75-.5 | 1. 1-.25 | 1. 1-.5 | 1. 1-.75 | 1. 1-1 |
| 1. 4 | 1. .25-.5 | 1. .5-.75 | 1. .5-1 | 1. .25-.75 | 1. .25-.25 | 1. .25-1 | 1. .75-1 | 1. .5-.5 | 1. 1-1 | 1. .5-.25 | 1. .75-.5 | 1. .75-.75 | 1. 1-.25 | 1. 1-.5 | 1. 1-.75 | 1. .75-.25 |
| 1. 5 | 1. .25-.75 | 1. .25-1 | 1. .25-.5 | 1. .5-1 | 1. .5-.75 | 1. 1-.75 | 1. .25-.25 | 1. .5-.25 | 1. .75-1 | 1. 1-.25 | 1. .5-.5 | 1. .75-.25 | 1. .75-.5 | 1. .75-.75 | 1. 1-.5 | 1. 1-1 |
| 1. 6 | 1. .5-.75 | 1. .5-1 | 1. .75-1 | 1. .25-.25 | 1. .25-.5 | 1. .25-1 | 1. .5-.25 | 1. 1-.25 | 1. .25-.75 | 1. .5-.5 | 1. .75-.25 | 1. .75-.75 | 1. .75-.5 | 1. 1-.5 | 1. 1-.75 | 1. 1-1 |
| 1. 7 | 1. .75-1 | 1. .5-.5 | 1. 1-1 | 1. .25-.5 | 1. .5-.25 | 1. .75-.75 | 1. .5-.75 | 1. .25-.25 | 1. .75-.25 | 1. .5-1 | 1. .25-.75 | 1. .25-1 | 1. .75-.5 | 1. 1-.75 | 1. 1-.25 | 1. 1-.5 |
| 1. 8 | 1. .5-.25 | 1. .75-.5 | 1. 1-.75 | 1. .25-.25 | 1. .5-.5 | 1. 1-.5 | 1. .75-.75 | 1. 1-1 | 1. .25-.5 | 1. .75-.25 | 1. 1-.25 | 1. .25-.75 | 1. .25-1 | 1. .5-.75 | 1. .5-1 | 1. .75-1 |
| 1. 9 | 1. .25-.5 | 1. .5-1 | 1. .25-.75 | 1. .75-1 | 1. .5-.75 | 1. .25-1 | 1. .75-.75 | 1. .25-.25 | 1. .5-.25 | 1. .75-.25 | 1. .75-.5 | 1. 1-.25 | 1. 1-1 | 1. .5-.5 | 1. 1-.5 | 1. 1-.75 |
| 1. 10 | 1. .25-.25 | 1. .75-.75 | 1. .5-.5 | 1. .5-.25 | 1. .75-.5 | 1. .25-.75 | 1. .25-1 | 1. .5-1 | 1. 1-.25 | 1. 1-.75 | 1. 1-.5 | 1. .75-1 | 1. .25-.5 | 1. .5-.75 | 1. 1-1 | 1. .75-.25 |
| 1. 11 | 1. .5-.25 | 1. 1-.75 | 1. .75-.5 | 1. .75-.25 | 1. .75-1 | 1. 1-.25 | 1. .5-1 | 1. .25-.75 | 1. .25-1 | 1. .25-.25 | 1. .25-.5 | 1. .5-.5 | 1. .5-.75 | 1. .75-.75 | 1. 1-.5 | 1. 1-1 |
| 1. 12 | 1. .25-.75 | 1. .25-1 | 1. .25-.5 | 1. .25-.25 | 1. .5-1 | 1. .5-.75 | 1. .75-1 | 1. .75-.75 | 1. 1-1 | 1. .5-.25 | 1. .5-.5 | 1. .75-.5 | 1. 1-.75 | 1. .75-.25 | 1. 1-.25 | 1. 1-.5 |
| 1. 13 | 1. .75-1 | 1. .25-.25 | 1. .5-.75 | 1. .5-1 | 1. .25-.5 | 1. 1-1 | 1. .25-.75 | 1. .5-.5 | 1. .5-.25 | 1. .75-.5 | 1. .25-1 | 1. .75-.75 | 1. .75-.25 | 1. 1-.25 | 1. 1-.5 | 1. 1-.75 |
| 1. 14 | 1. .5-.25 | 1. 1-.5 | 1. .75-.25 | 1. 1-.75 | 1. 1-1 | 1. .75-.5 | 1. .75-.75 | 1. .5-.5 | 1. .25-.5 | 1. .25-.75 | 1. .5-.75 | 1. .75-1 | 1. 1-.25 | 1. .25-.25 | 1. .25-1 | 1. .5-1 |
| 1. 15 | 1. .25-.25 | 1. .5-.5 | 1. .75-1 | 1. .5-.75 | 1. .5-1 | 1. .25-1 | 1. .25-.5 | 1. .25-.75 | 1. .75-.75 | 1. .5-.25 | 1. .75-.25 | 1. .75-.5 | 1. 1-1 | 1. 1-.25 | 1. 1-.5 | 1. 1-.75 |
| 1. 16 | 1. .5-.75 | 1. .5-1 | 1. .25-.25 | 1. .25-.5 | 1. .25-1 | 1. .25-.75 | 1. .5-.5 | 1. .75-.5 | 1. .5-.25 | 1. .75-1 | 1. .75-.25 | 1. .75-.75 | 1. 1-.25 | 1. 1-.5 | 1. 1-.75 | 1. 1-1 |
| 1. 17 | 1. .25-.75 | 1. .25-.5 | 1. .25-.25 | 1. .25-1 | 1. .5-1 | 1. .5-.25 | 1. .5-.5 | 1. .5-.75 | 1. .75-.25 | 1. .75-.5 | 1. .75-.75 | 1. .75-1 | 1. 1-.25 | 1. 1-.5 | 1. 1-1 | 1. 1-.75 |
| 1. 18 | 1. .5-1 | 1. .5-.75 | 1. .25-.5 | 1. .75-1 | 1. .25-.25 | 1. .5-.5 | 1. .25-.75 | 1. 1-1 | 1. .25-1 | 1. .5-.25 | 1. .75-.75 | 1. .75-.25 | 1. .75-.5 | 1. 1-.25 | 1. 1-.5 | 1. 1-.75 |
| 1. 19 | 1. .5-1 | 1. .25-.5 | 1. .25-.75 | 1. .5-.75 | 1. .75-1 | 1. .25-1 | 1. .5-.5 | 1. .5-.25 | 1. .25-.25 | 1. .75-.25 | 1. .75-.5 | 1. .75-.75 | 1. 1-.25 | 1. 1-.5 | 1. 1-.75 | 1. 1-1 |
| 1. 20 | 1. .25-.25 | 1. 1-1 | 1. .75-.75 | 1. 1-.75 | 1. .5-.5 | 1. .75-1 | 1. .75-.5 | 1. .25-.5 | 1. .25-.75 | 1. .25-1 | 1. .5-.75 | 1. .5-1 | 1. .5-.25 | 1. .75-.25 | 1. 1-.5 | 1. 1-.25 |
| 1. 21 | 1. .25-.25 | 1. 1-1 | 1. .75-1 | 1. .25-.5 | 1. .5-.25 | 1. .5-.75 | 1. .25-.75 | 1. .5-1 | 1. .75-.75 | 1. .75-.25 | 1. .25-1 | 1. .5-.5 | 1. .75-.5 | 1. 1-.25 | 1. 1-.75 | 1. 1-.5 |
| 1. 22 | 1. .5-.75 | 1. .5-1 | 1. .75-1 | 1. .25-.25 | 1. .25-.5 | 1. .25-1 | 1. .25-.75 | 1. .5-.25 | 1. .5-.5 | 1. .75-.75 | 1. 1-1 | 1. .75-.25 | 1. .75-.5 | 1. 1-.25 | 1. 1-.5 | 1. 1-.75 |
| 1. 23 | 1. .75-.25 | 1. 1-.5 | 1. 1-.25 | 1. 1-.75 | 1. .25-.5 | 1. .75-.5 | 1. .5-.25 | 1. .25-.75 | 1. .25-1 | 1. .5-.5 | 1. .5-.75 | 1. .5-1 | 1. .75-.75 | 1. .75-1 | 1. 1-1 | 1. .25-.25 |
| 1. 24 | 1. .25-1 | 1. .25-.5 | 1. .25-.75 | 1. .5-.75 | 1. .5-1 | 1. .75-.25 | 1. .25-.25 | 1. .75-1 | 1. .5-.25 | 1. .5-.5 | 1. .75-.5 | 1. .75-.75 | 1. 1-.25 | 1. 1-.5 | 1. 1-.75 | 1. 1-1 |
| 1. 25 | 1. .75-.5 | 1. .5-.25 | 1. 1-.75 | 1. 1-1 | 1. 1-.5 | 1. .25-.25 | 1. .75-.75 | 1. .5-.5 | 1. 1-.25 | 1. .25-.5 | 1. .75-.25 | 1. .75-1 | 1. .25-.75 | 1. .25-1 | 1. .5-.75 | 1. .5-1 |
| 1. 26 | 1. .25-.25 | 1. .75-.75 | 1. .5-.5 | 1. 1-1 | 1. .75-.25 | 1. .25-.5 | 1. .5-.75 | 1. .25-.75 | 1. .5-.25 | 1. .75-.5 | 1. .75-1 | 1. 1-.75 | 1. .25-1 | 1. .5-1 | 1. 1-.25 | 1. 1-.5 |
| 1. 27 | 1. 1-.75 | 1. .25-.25 | 1. .75-.5 | 1. 1-1 | 1. .75-.75 | 1. .5-.25 | 1. .5-.5 | 1. .75-.25 | 1. .25-.5 | 1. .25-.75 | 1. 1-.5 | 1. .5-1 | 1. .25-1 | 1. .75-1 | 1. 1-.25 | 1. .5-.75 |
| 1. 28 | 1. .5-.75 | 1. .25-.25 | 1. .75-1 | 1. .5-1 | 1. .5-.5 | 1. .25-.5 | 1. .25-1 | 1. .25-.75 | 1. .75-.75 | 1. 1-.75 | 1. .5-.25 | 1. .75-.25 | 1. .75-.5 | 1. 1-.25 | 1. 1-.5 | 1. 1-1 |
| 1. 29 | 1. .25-.75 | 1. .5-1 | 1. .25-1 | 1. .75-1 | 1. .25-.5 | 1. .5-.75 | 1. .25-.25 | 1. .5-.25 | 1. .5-.5 | 1. .75-.25 | 1. .75-.5 | 1. .75-.75 | 1. 1-.25 | 1. 1-.75 | 1. 1-1 | 1. 1-.5 |
| 1. 30 | 1. 1-.75 | 1. .75-.5 | 1. .75-.25 | 1. 1-.5 | 1. .5-.25 | 1. 1-.25 | 1. .25-.25 | 1. .25-.5 | 1. .25-.75 | 1. .25-1 | 1. .5-.5 | 1. .5-.75 | 1. .75-.75 | 1. .75-1 | 1. 1-1 | 1. .5-1 |
| 1. 31 | 1. .75-.25 | 1. .25-.25 | 1. .25-.75 | 1. .25-1 | 1. .5-.25 | 1. .5-.5 | 1. .5-.75 | 1. .5-1 | 1. .75-.75 | 1. .75-1 | 1. 1-.25 | 1. 1-1 | 1. .25-.5 | 1. .75-.5 | 1. 1-.5 | 1. 1-.75 |
| 1. 32 | 1. .25-.5 | 1. .75-1 | 1. .5-.75 | 1. .5-1 | 1. 1-1 | 1. .25-.75 | 1. .75-.75 | 1. .25-.25 | 1. 1-.75 | 1. .25-1 | 1. .5-.5 | 1. .5-.25 | 1. .75-.25 | 1. .75-.5 | 1. 1-.25 | 1. 1-.5 |
| 1. 33 | 1. .25-1 | 1. .25-.75 | 1. .25-.5 | 1. .75-1 | 1. .25-.25 | 1. .75-.25 | 1. .5-1 | 1. .5-.75 | 1. .5-.25 | 1. .5-.5 | 1. .75-.5 | 1. .75-.75 | 1. 1-.25 | 1. 1-.5 | 1. 1-.75 | 1. 1-1 |
| 1. 34 | 1. .5-1 | 1. .5-.75 | 1. .75-1 | 1. .25-.5 | 1. .25-.25 | 1. .25-.75 | 1. .25-1 | 1. .5-.5 | 1. .75-.75 | 1. 1-.25 | 1. .5-.25 | 1. .75-.25 | 1. .75-.5 | 1. 1-.5 | 1. 1-.75 | 1. 1-1 |
| 1. 35 | 1. .25-1 | 1. .5-1 | 1. .75-1 | 1. .25-.75 | 1. .5-.75 | 1. .25-.5 | 1. .75-.75 | 1. .75-.5 | 1. .25-.25 | 1. .75-.25 | 1. .5-.25 | 1. .5-.5 | 1. 1-.25 | 1. 1-.5 | 1. 1-.75 | 1. 1-1 |
| 1. 36 | 1. .25-.25 | 1. .5-.5 | 1. .75-1 | 1. .25-.5 | 1. .25-.75 | 1. .5-1 | 1. .25-1 | 1. .5-.75 | 1. 1-.75 | 1. .75-.75 | 1. .5-.25 | 1. 1-1 | 1. .75-.25 | 1. .75-.5 | 1. 1-.25 | 1. 1-.5 |
| 1. 37 | 1. 1-1 | 1. .5-.75 | 1. .75-.75 | 1. .5-.5 | 1. .75-1 | 1. .5-1 | 1. .25-.5 | 1. .25-.25 | 1. .25-.75 | 1. .25-1 | 1. .75-.25 | 1. .5-.25 | 1. .75-.5 | 1. 1-.25 | 1. 1-.5 | 1. 1-.75 |
| 1. 38 | 1. .25-.75 | 1. .5-1 | 1. .25-.5 | 1. .75-1 | 1. .5-.75 | 1. .25-.25 | 1. .5-.5 | 1. 1-1 | 1. .25-1 | 1. .75-.25 | 1. .75-.5 | 1. .75-.75 | 1. .5-.25 | 1. 1-.25 | 1. 1-.5 | 1. 1-.75 |
| 1. 39 | 1. .25-.25 | 1. .75-1 | 1. .5-.75 | 1. .25-.5 | 1. .5-.5 | 1. .5-1 | 1. .25-.75 | 1. .25-1 | 1. .5-.25 | 1. .75-.25 | 1. .75-.5 | 1. .75-.75 | 1. 1-.25 | 1. 1-.5 | 1. 1-.75 | 1. 1-1 |
| 1. 40 | 1. 1-1 | 1. .25-.25 | 1. .5-.5 | 1. .75-.75 | 1. .5-.25 | 1. .75-.5 | 1. 1-.75 | 1. .25-1 | 1. .5-.75 | 1. .5-1 | 1. .75-.25 | 1. .25-.5 | 1. .25-.75 | 1. .75-1 | 1. 1-.25 | 1. 1-.5 |

1. **S3 Does anticipation of punishment lead to an increased MFT power?**
2. To investigate this possibility, we performed following analyses:
3. MFT power as function of punishment probability
4. We investigated whether MFT power increases monotonically with *punishment* probability regardless of reward. We proceeded with assumption that a high punishment probability leads to high anticipation of punishment. We calculated MFT power at FCz channel for four punishment conditions in a time window of 500-1000 ms post stimulus onset, ignoring reward probability. As can be seen in the figure below, we find that MFT power does not increase monotonically with increase in punishment probability.
5.
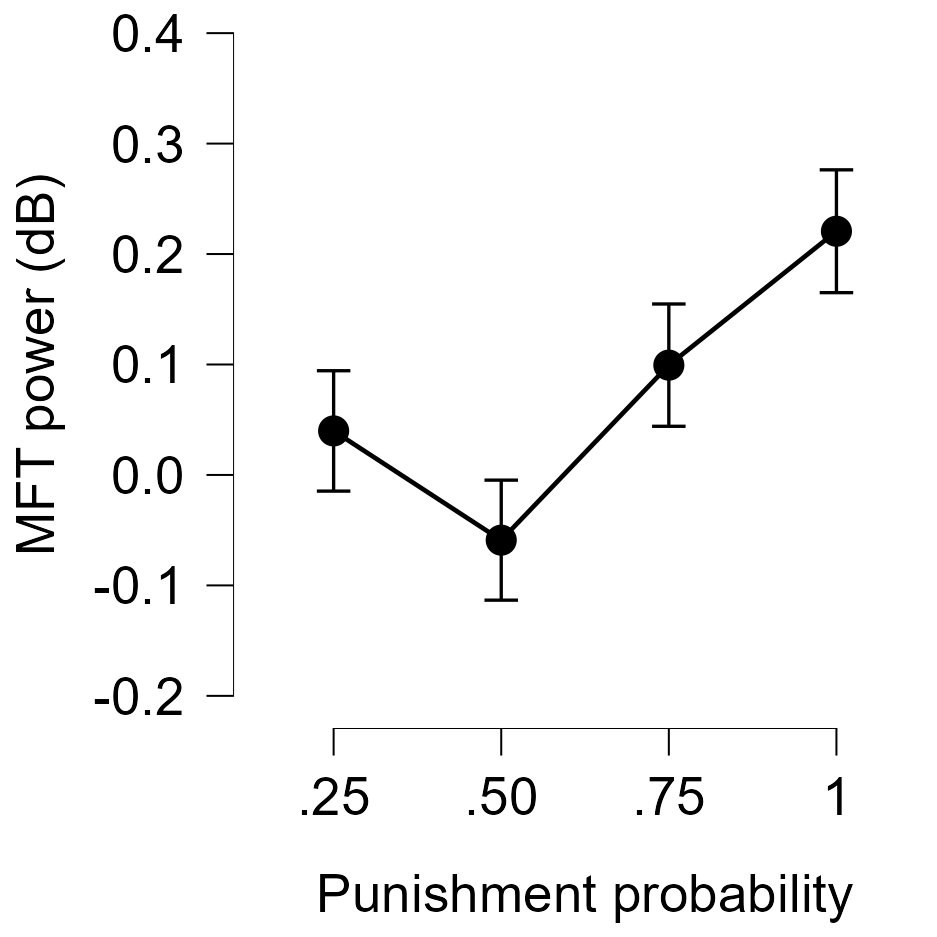

6. Computing MFT power separately for reward-accept and reward-reject trials:
7. We performed our MFT power analysis after dividing our trials in two groups: a) trials where participants rejected the bet, i.e., they rejected reward and punishment. Such trials have conflict induced by stimulus, but do not have anticipation/expectation of reward/punishment. b) trials where participant accepted the bet. Such trials have both stimulus conflict as well as anticipation/expectation of reward/punishment. In the next step, we computed MFT power of maximum and minimum conflict trials, conflict defined based on BCI scores. We find that MFT power still increases post stimulus onset in those trials where participants rejected reward/punishment. This result suggests that increase in MFT power is not driven by anticipation of punishment.
8.
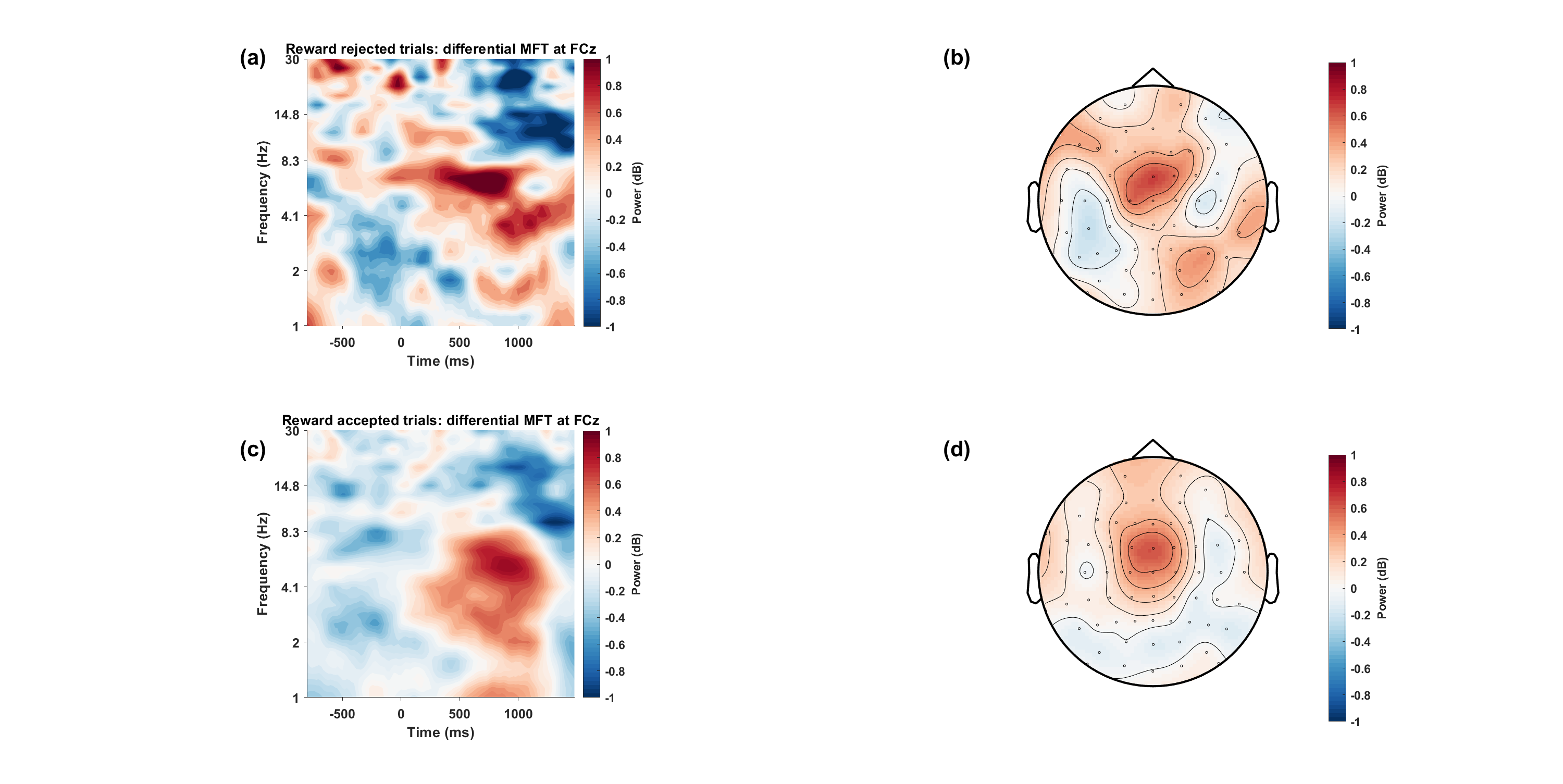

9. Contrasting trials with punishment+conflict with conflict only
10. As an additional analysis, we contrasted trials with high reward + high threat (dominated by anticipation of punishment and conflict) vs. high reward + low threat (dominated by conflict only). For this purpose, we chose trials of condition 1–1 and 1–.25, respectively. If we subtract these conditions, what we get would be the effect of punishment. In the next step, we calculated differential MFT activity between these conditions (panel c below figure). We do not find a significant increase in MFT power in two conditions. We computed point estimate of MFT power using a time window of 500-1000 ms post stimulus onset and performed a t-test. The difference was not significant, *t*(39) = 0.77, *p* = 0.44. This results further suggest that anticipation of punishment did not modulate increase in MFT power.
11.
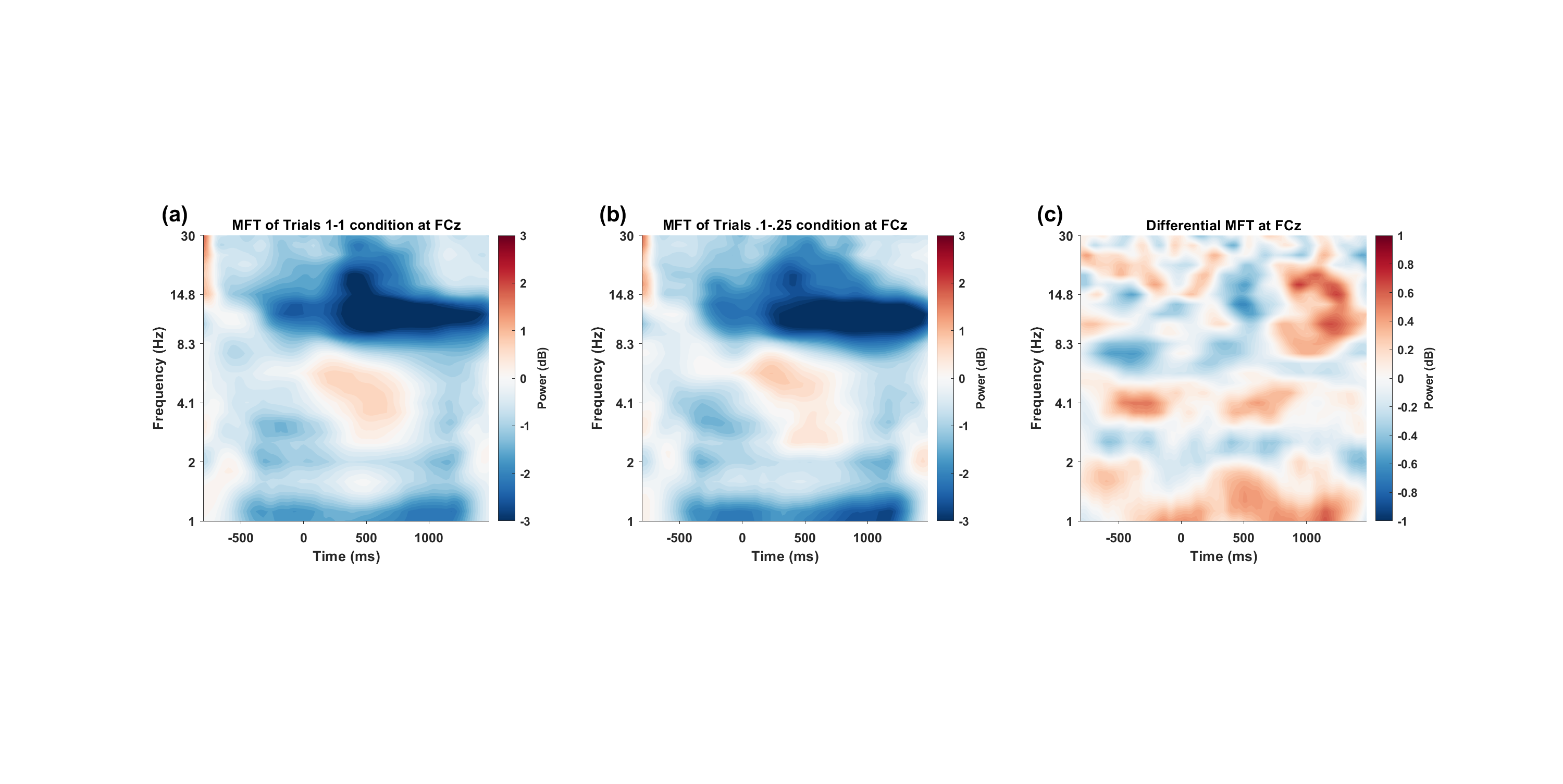

12. Together, these results points that an increase in MFT power in our design is mostly driven by stimulus conflict.

**S4. Does longer RT lead to an increased MFT power?**

We investigated the idea whether longer reaction time or related motor planning leads to enhanced MFT in maximum conflict conditions. We did following analyses:

Computing MFT from an early time window:

In our data, MFT power in the maximum conflict condition began to increase around 200 ms after stimulus onset compared to the minimum conflict condition, well before any responses were made. This suggests that the observed theta increase reflects stimulus-induced conflict rather than response-related processes. If reaction times contributed substantially, they should not influence theta power prior to response execution. Conversely, a differential increase in stimulus-locked theta before the earliest responses would indicate a genuine conflict-related effect. To test this, we computed MFT power in an early window of 300–500 ms post-stimulus, rather than the broader 500–1000 ms window identified by cluster-based permutation tests. Importantly, this early window lies entirely before the average response time in even the fastest condition (1–0.25, mean RT = 559 ms). Results showed that MFT power was significantly higher for maximum (M = 0.75 dB, SD = 1.20) than minimum conflict (M = 0.36 dB, SD = 0.98), t(39) = 3.15, p = .003, d = 0.34. These findings suggest that the MFT increase is primarily stimulus-driven, with minimal contribution from response times or motor preparation.


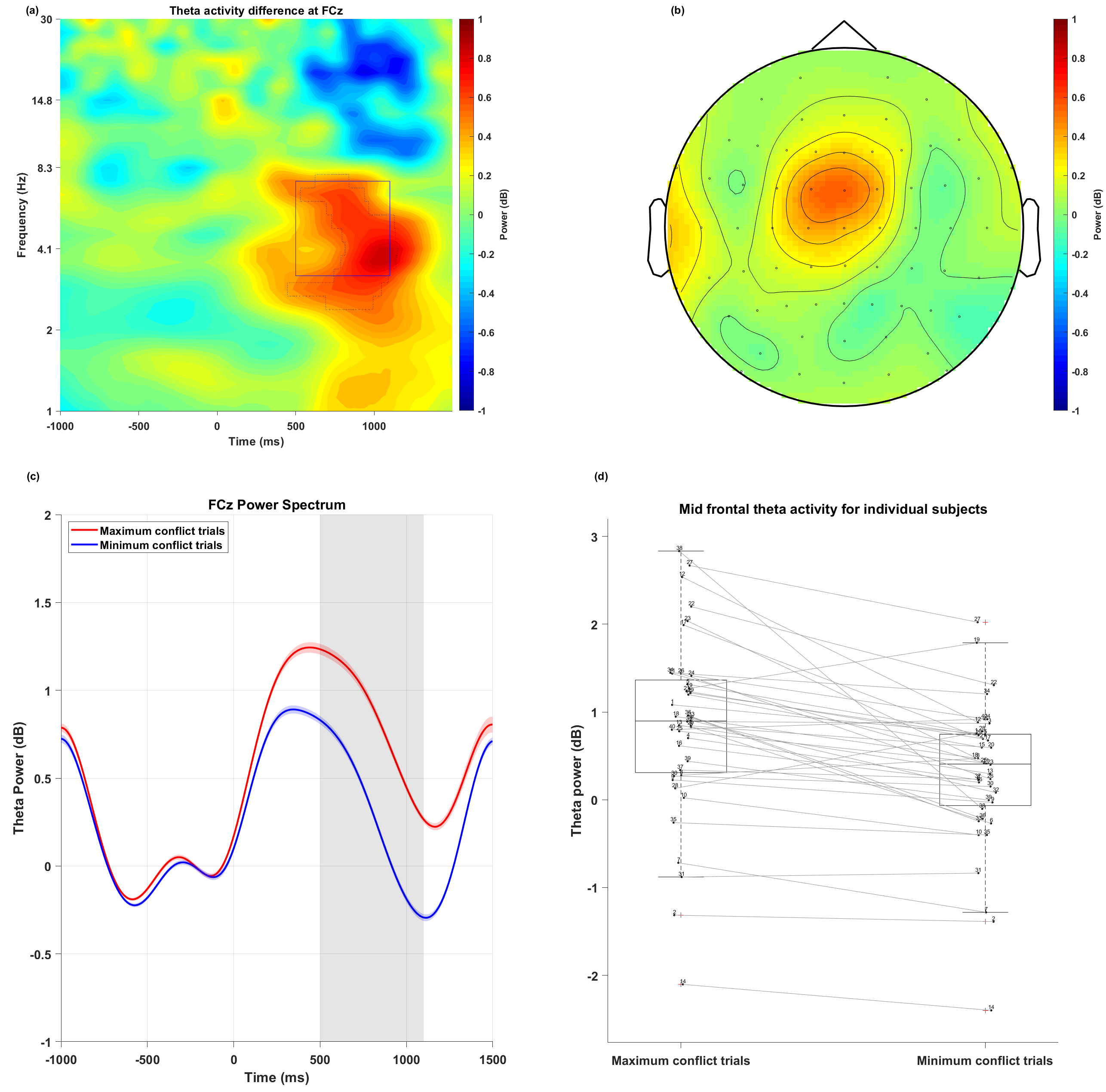


Correlation of RT and theta:

We further examined whether the increase in MFT power was associated with reaction time by correlating the difference in theta power between maximum and minimum conflict conditions with the corresponding RT difference. This analysis revealed no significant relationship, r = 0.09, p = .54, indicating that the observed increase in theta cannot be explained by slower responses and is therefore unlikely to reflect mere reaction time effects.


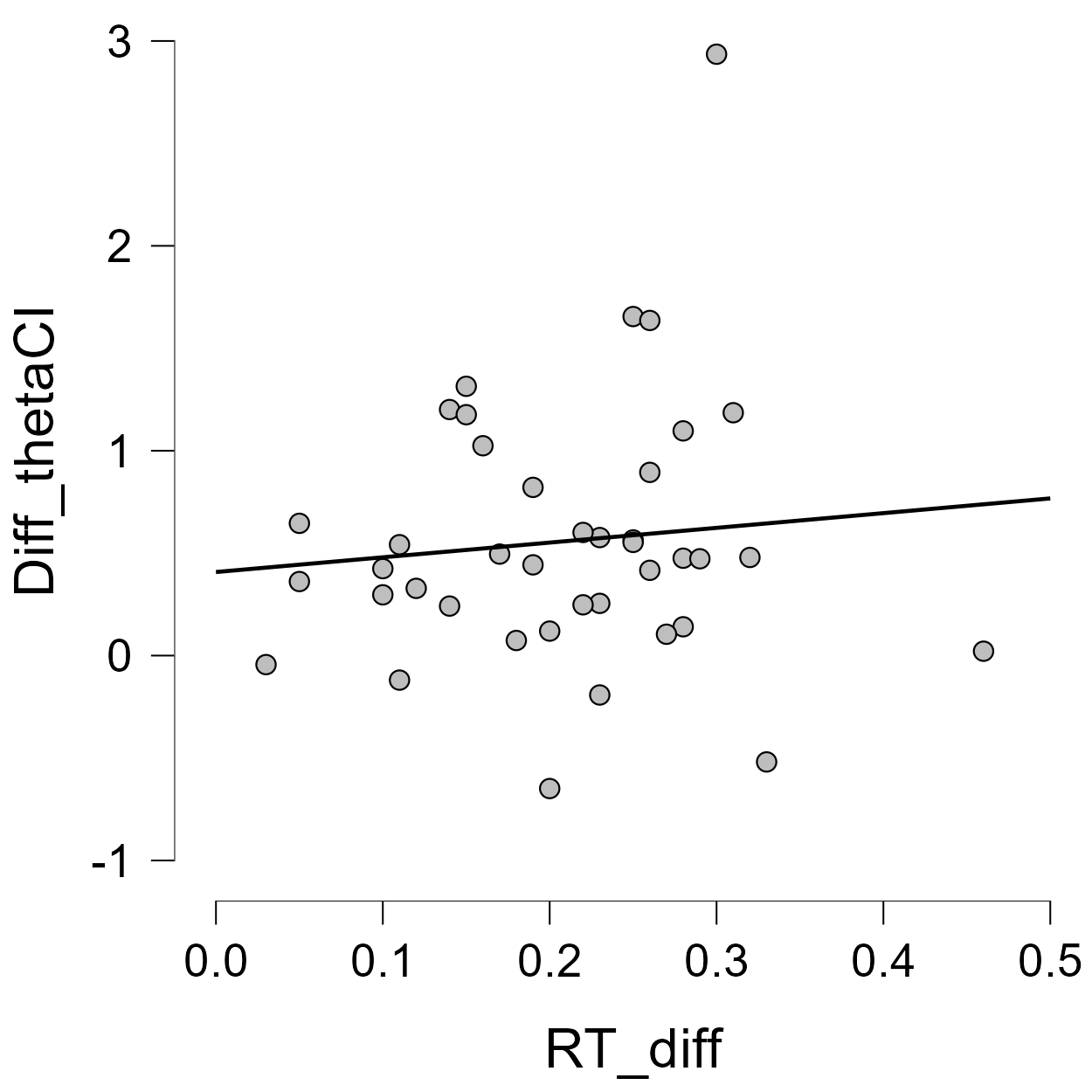


Computing MFT for high and low RT trials irrespective of conflict:

To directly examine whether the increase in MFT power for maximum conflict trials was driven by longer response times, we conducted a median-split analysis. Within each conflict condition (maximum and minimum), trials were divided into high-RT and low-RT subsets. We then merged maximum–high RT trials with minimum–high RT trials, and maximum–low RT trials with minimum–low RT trials, resulting in two RT-based conditions (high RT and low RT), irrespective of conflict. Permutation-cluster correction revealed no significant clusters in the differential time–frequency plots at FCz (see below figure). Furthermore, when focusing on the a priori theta window (500–1000 ms post-stimulus), we found no significant difference in MFT power between high- and low-RT trials, *t*(39) = 1.85, *p* = .07, *d* = 0.06. These results further support the interpretation that increased MFT power reflects conflict-related cognitive processing rather than being a simple consequence of longer response times.


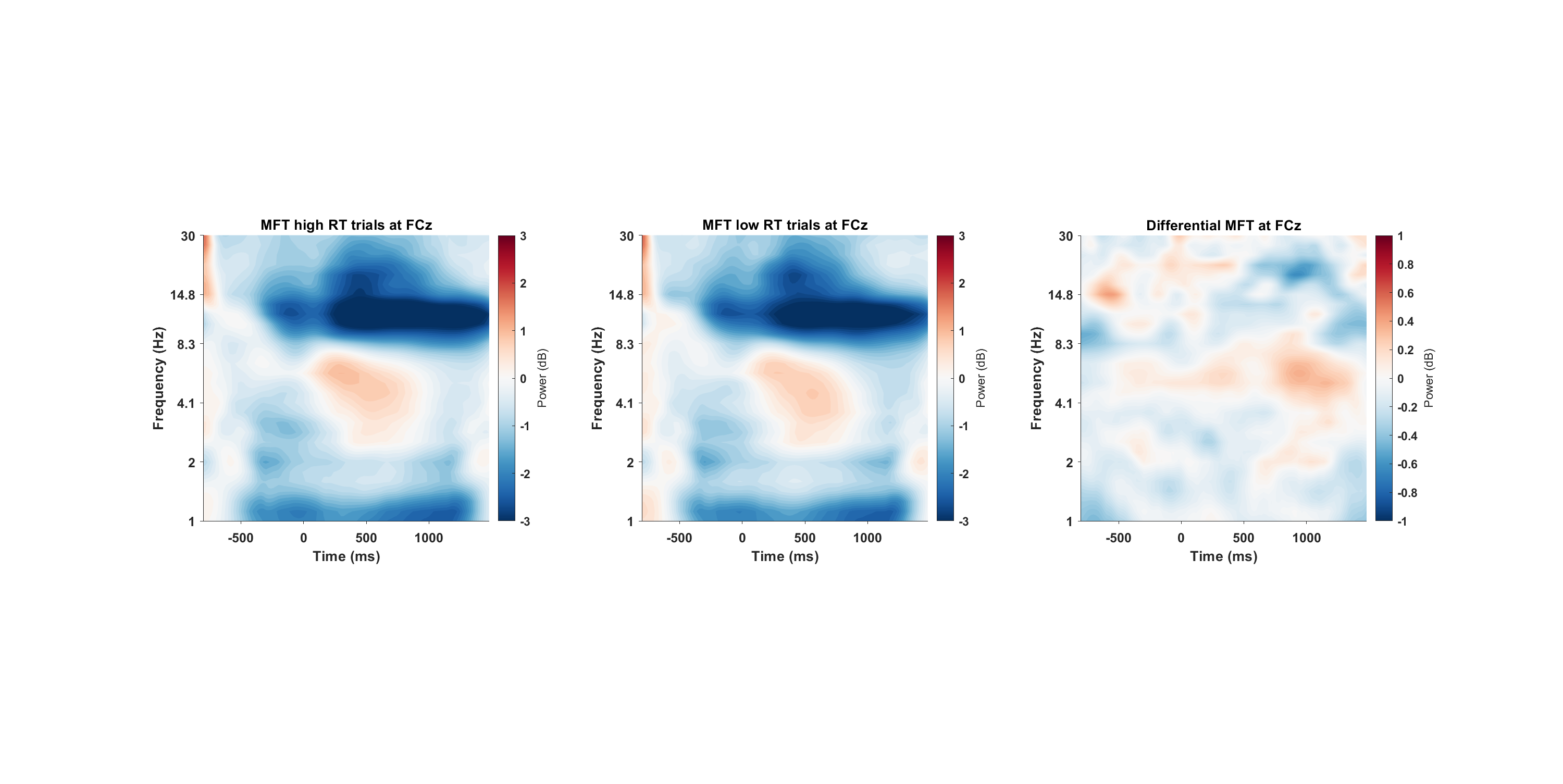


1. **S5 BIS-MFT correlation is not driven by an increase in reaction time**
2. We find no correlation (all correlation, r < 0.03) between BIS score and reaction time in high conflict or low conflict trials (left and middle figure below). We also do not find that participants with higher BIS score show more RT difference (right figure below). Therefore, we can say that the correlation between differential theta and BIS score is not driven by reaction time.
3.
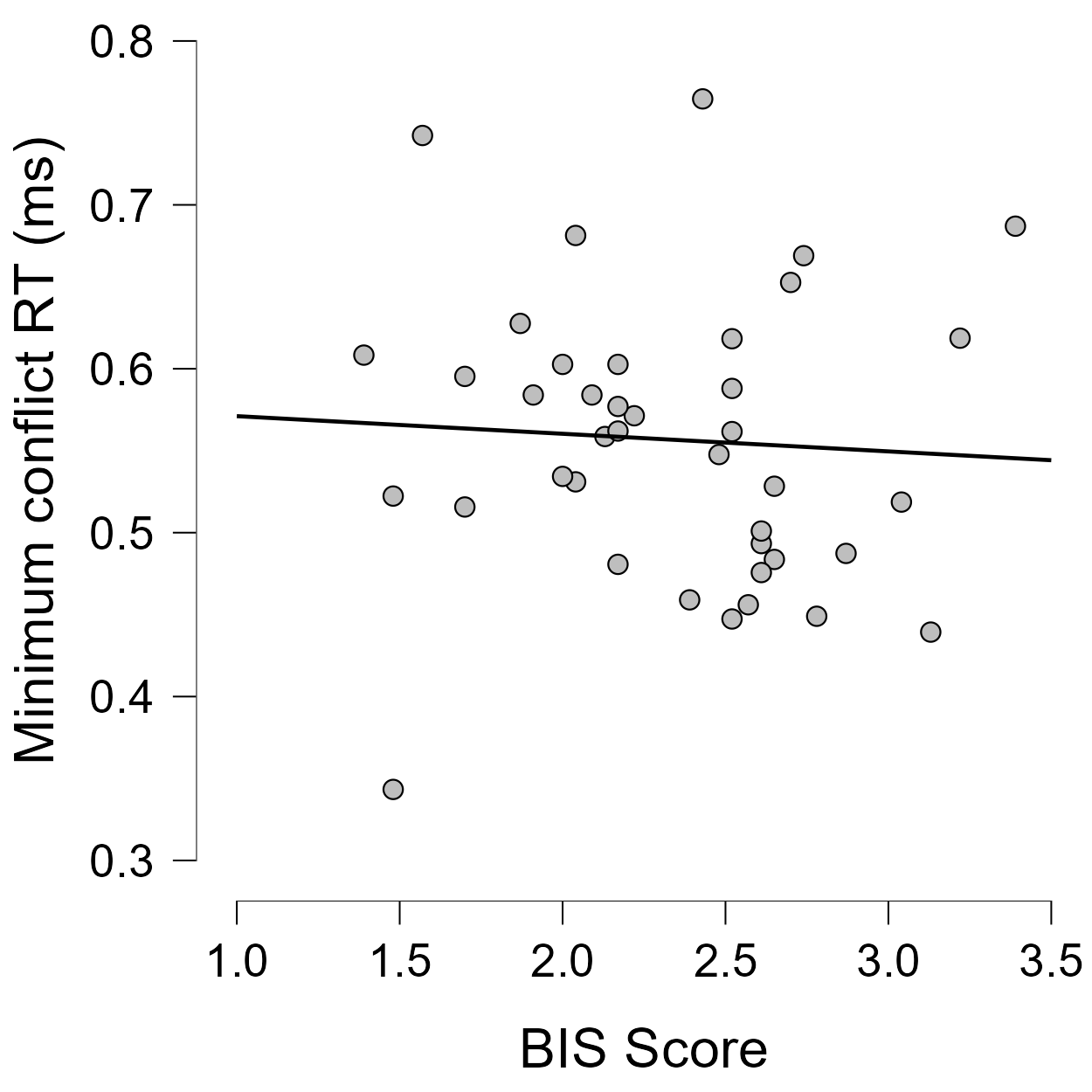

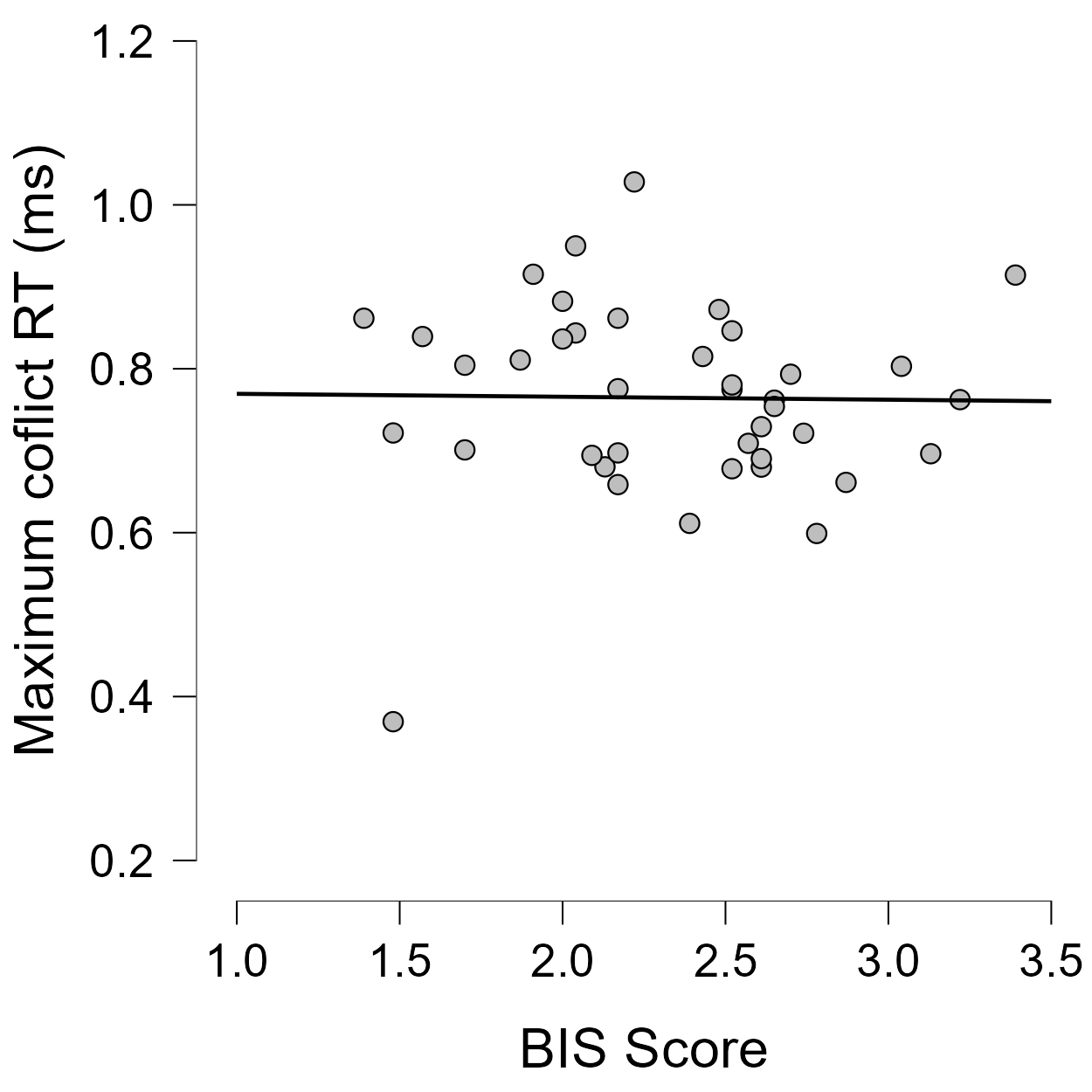

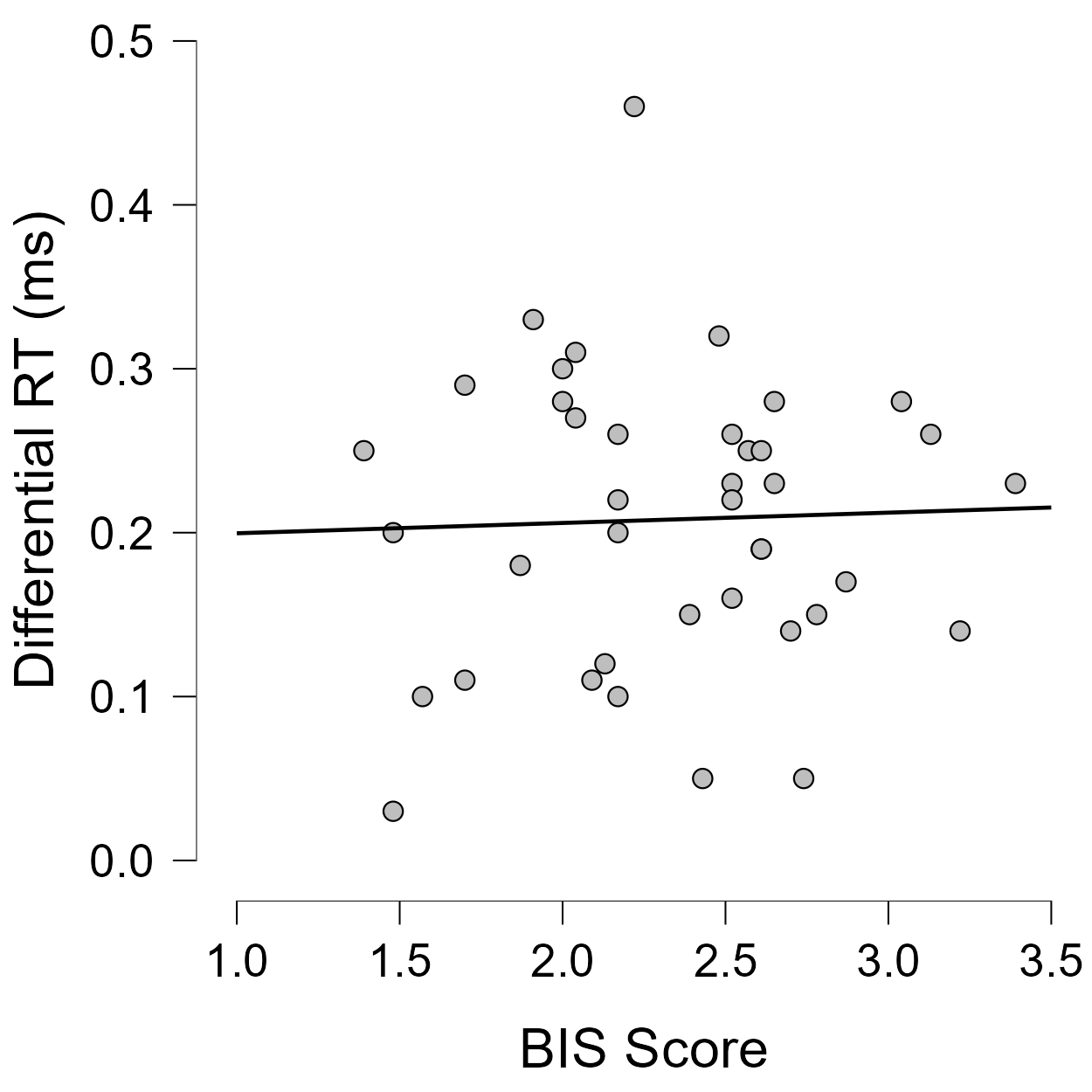


**S6: Best Rescorla Wagner model for each participants (highlighted in green colour)**

1. We applied computational modelling to find out how participants exactly weigh reward and punishment while deciding in our task. For this purposes, we compared fit of four models, all of which are variations of a standard Rescorla-Wagner model (Moughrabi et al., 2022): 1) a reward only model (R model), positing that participants ignore the punishment outcomes and only use information about reward outcomes to guide decisions, 2) a punishment only model (P model), positing that participants only use information about punishment outcomes to guide decisions, 3) a reward and punishment model using a single learning rate (RP single model), positing that participants integrate reward and punishment expectations into a combined total value and using one learning rate for both outcomes, and 4) a similar reward and punishment model but using separate learning rates for rewards and punishments (RP double model). We chose Rescorla-Wagner models as they allow to model reward and punishment influences, and their interactions, simultaneously.
2. The R model updates expected reward value *VR* of a chosen option (i.e. reward expectation), based on the magnitude of the prediction error *δ*_R_ (observed outcome – V), scaled by a learning rate *α* (ranging from 0 to 1) in trial-to-trial (t to t+1) manner: *VC: VR*_t+1_ = *VR*_t_ + *δ*_R_ × *α_R_*. Similarly, the P model updates the expected punishment value of a chosen option (i.e. punishment expectation), *VP*, based on the magnitude of the prediction error, *δ*_P_ (observed outcome – V), scaled by a learning rate *α_p_* (ranging from 0 to 1): *VC: VP*_t+1_ = *VP*_t_ + *δ*_P_ × *α_P_*.
3. The RP single model was combination of R model and P model, where the same learning rate was used for both the VR and VP updating (*α_R_* and *α_P_* were same). The separate expectations for VR and VP were then integrated into a combined value, VC, using an individually varying and scalar policy parameter, π, that represents an individual’s preference for reward approach vs punishment avoidance: *VC*_t+1_ = *VR*_t_ (1- *π*) + (1- *VP*_t_ ) *π.* A value of 0 for *π* indicates a full preference for reward approach with no preference for punishment avoidance while making a decision; a value of 1 indicates a full preference for punishment avoidance with no preference for reward approach; an intermediate value from 0 to 1 indicates an increase in punishment weight and decrease in reward weight. The RP double model was identical to the RP single model, except a separate learning rate was used for reward, *α_R_*, and punishment, *α_P_*, outcomes. For each model, a softmax function transformed expected value (*VC*) into action probabilities by using an exploration / exploitation *β* parameter. A higher softmax *β* represents a tendency to exploit high value responses / respond more consistently; a lower softmax *β* represents a tendency to explore lower value options / respond more inconsistently.
4. Accordingly, the R and P models had 2 free parameters (*α*, *β*), the RP single model had 3 free parameters (*α*, *β, π*), and the RP double model had 4 free parameters (*αR αP*, *β, π*). Model fitting and parameter optimization were conducted using **maximum likelihood estimation (MLE)** with a **constrained optimization** approach. Models were compared using Akaike Information Criterion (AIC) comparison since R, P, and RP single models are nested within the RP double mode.
5. Although a Rescorla–Wagner structure was implemented, the reward and punishment probabilities were explicitly provided on each trial. Therefore, we did not treat this model as a learning model but rather as a value integration model. In this framework, the expected reward (VR) and expected punishment (VT) on each trial were set to the objective probabilities presented to participants. The free parameters (e.g., integration parameter π, and inverse temperature β) therefore quantify how participants combined the known reward and punishment information into a subjective value signal that guided choice behavior. This approach allows estimation of individual differences in how strongly participants weighted reward versus punishment information, without assuming any learning from feedback across trials.
6. **Results**
7. For 70% (28 out of 40) of participants, the RP single model yielded the best prediction regarding participant response to given trial. This suggests that most participants integrated reward and punishment value into a combined total value according to an individually varying policy parameter, which was highly predictive of decisions to approach reward vs avoid punishment. For participants with R model as best model, the difference in AIC between R model and RP single model was meagre. Therefore, for simplicity we extracted the parameter *π* for all participants from the third model, the RP single model. The parameter *π* correlated with reward rejection rate across all conditions, *r* = -0.94, *p* < .001.

**S7: Figures based on other approaches**


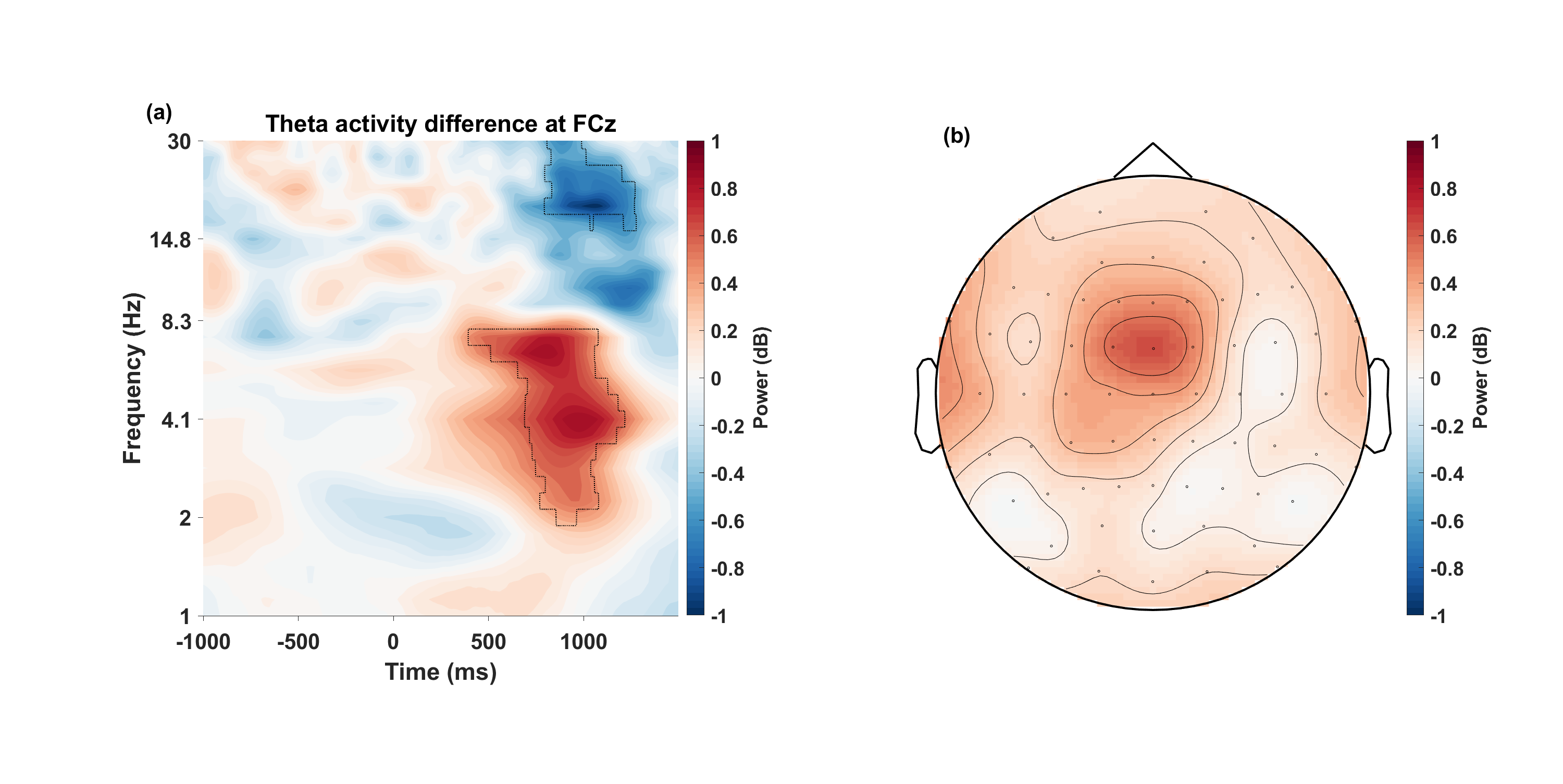


Individualised RT based approach


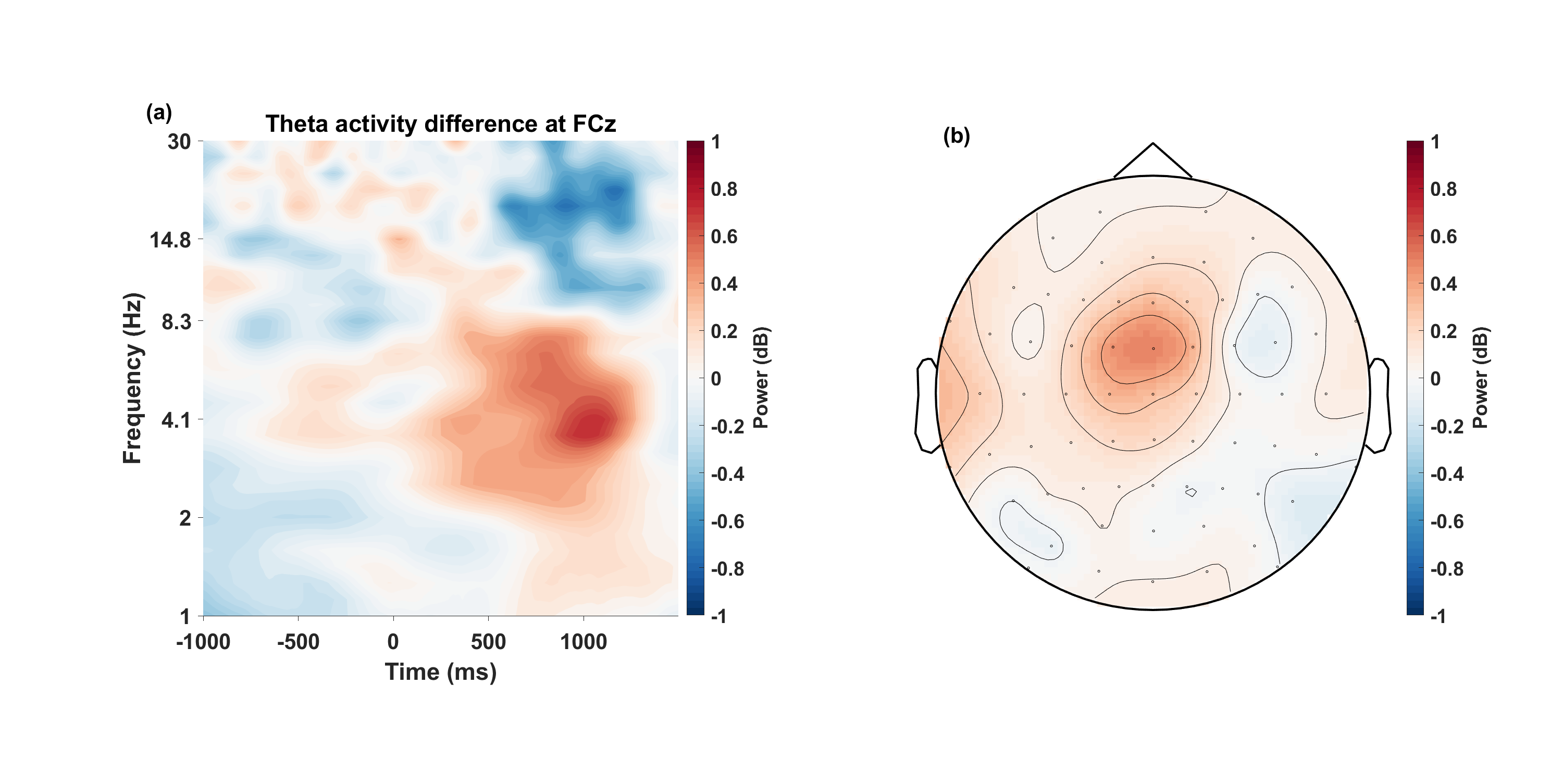


Individualised rate based approach


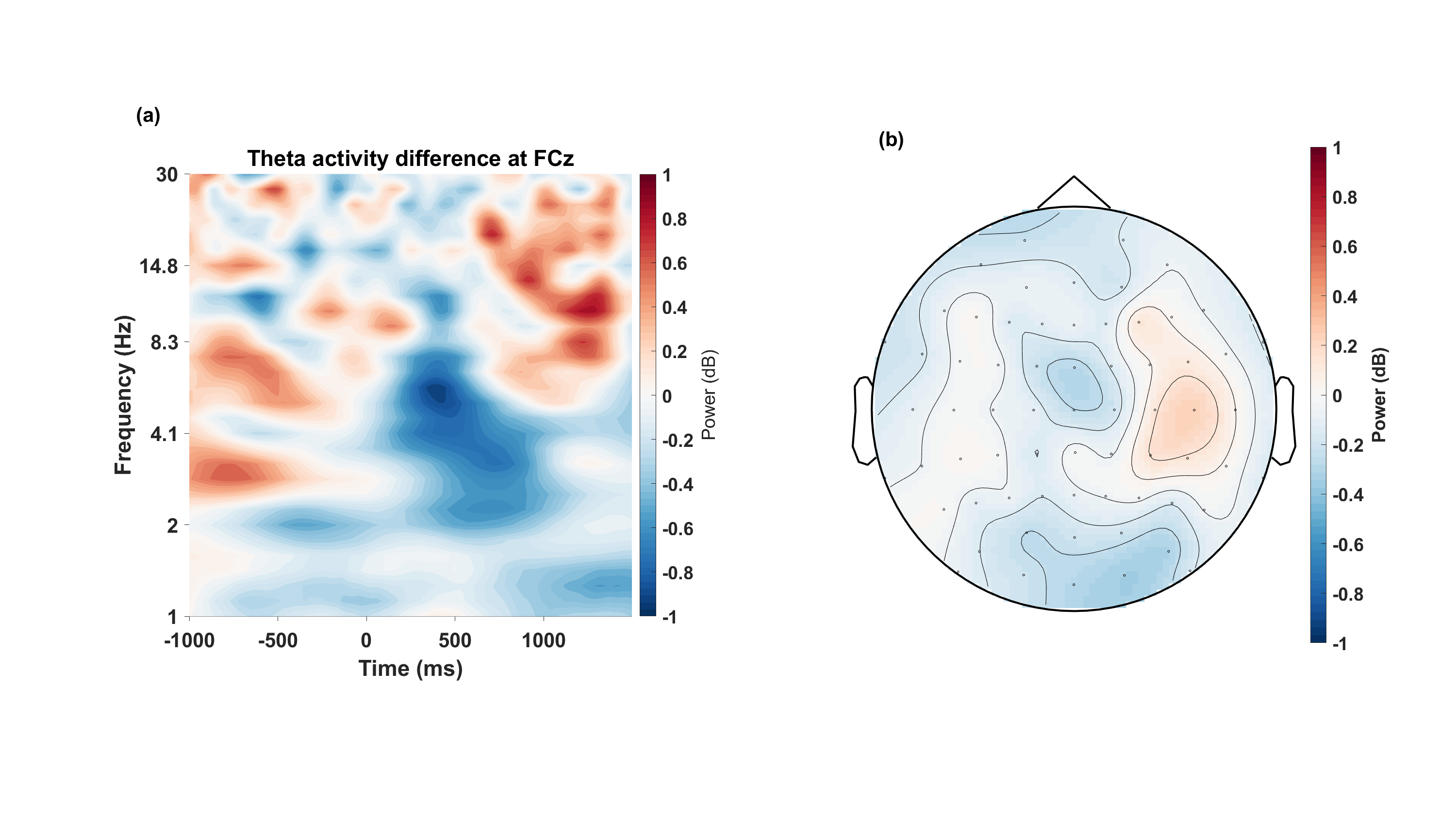


Uniform approach: minimum AAC 100% -25%

1. **S8: Catch trials**
2. In our experiment, we also wanted to ensure that participants were paying attention to probabilities of reward and punishment. For this purposes, we delivered catch trials after feedback and before inter-trial interval on three random trials (approximate 5%) in every block. On these trials, we presented two bars on top and bottom part of screen and asked participant to discriminate which of these two bars was the bar they saw on the previous trial. From the previous trial, we randomly chose one of the two bars target. For current catch trial, we coupled this bar from previous trial with a bar from same category (green: reward, red: threat) but differing in 25% probability. For example, if a threat meter of 50% probability appeared on pervious trial and was randomly chosen, we added a threat meter of either 25% or 75% probability, randomly chosen, and presented at the above or below the centre of screen, randomly. Between these two meters, the text read, “Which of the two bars was present on the last trial? Press top arrow key for top bar and down arrow key for bottom bar.” The study instructions sheet had no information about catch trials and we also did not put much emphasis on them to ensure participants’ main task remains deciding to chose reward or not. For all participants, the catch trial accuracy was greater than 50% chance level, *M* = 74.47, *SD* = 11.33. A one sample t-test showed that catch accuracy was significantly higher than chance level, *t*(39) = 13.65, *p* < .001, *d* = 2.16. This result indicates that participants were actively paying attention to reward and threat probabilities.
